# Defective Cystic Fibrosis Transmembrane Conductance Regulator Accelerates Skeletal Muscle Aging by Impairing Autophagy/Myogenesis

**DOI:** 10.1002/jcsm.13708

**Published:** 2025-01-29

**Authors:** Ziyi Chen, Jiankun Xu, Peijie Hu, Wanting Du, Junjiang Chen, Xiaotian Zhang, Wei Zhou, Jiayang Gao, Yuantao Zhang, Bingyang Dai, Guangshuai Nie, Jun Hu, Liangbin Zhou, Shunxiang Xu, Hisao Chang Chan, Wing‐hoi Cheung, Ye Chun Ruan, Ling Qin

**Affiliations:** ^1^ Musculoskeletal Research Laboratory, Department of Orthopedics & Traumatology The Chinese University of Hong Kong Hong Kong China; ^2^ Department of Biomedical Engineering, Faculty of Engineering The Hong Kong Polytechnic University Hong Kong China; ^3^ State Key Laboratory of Respiratory Disease for Allergy Shenzhen Key Laboratory of Allergy & Immunology School of Medicine Shenzhen University Shenzhen China; ^4^ School of Life Sciences, Centre for Cell & Developmental Biology and State Key Laboratory of Agrobiotechnology The Chinese University of Hong Kong Hong Kong China; ^5^ Orthopaedic Research Centre, Department of Orthopaedics The First Affiliated Hospital of Shantou University Medical College Shantou China; ^6^ Epithelial Cell Biology Research Centre, School of Biomedical Sciences, Faculty of Medicine The Chinese University of Hong Kong Hong Kong China

**Keywords:** autophagy, CFTR, CFTR‐modulator, mitochondria, myogenesis, skeletal muscle

## Abstract

**Background:**

Regenerative capacity of skeletal muscles decreases with age. Deficiency in cystic fibrosis transmembrane conductance regulator (CFTR) is associated with skeletal muscle weakness as well as epithelial cell senescence. However, whether and how CFTR plays a role in skeletal muscle regeneration and aging were unclear.

**Methods:**

Vastus lateralis biopsy samples from male and female human subjects (*n* = 23) of 7‐ to 86‐year‐old and gastrocnemii tissues from mice of 4‐ to 29‐month‐old were examined for CFTR expression. Skeletal muscle tissues or cultured myoblasts from mice carrying CFTR mutation (DF508) at 4‐ to 18‐month‐old were used for assessment of muscle mass, contractile force and regenerative capacity as well as myogenic and autophagy signalling. Overexpression of LC3‐*β*, an autophagy mediator, was conducted to reverse myogenic defects in DF508 myoblasts. Adenoviruses containing CFTR gene or pharmaceuticals that enhance CFTR (VX809) were locally injected into the gastrocnemius or femoris quadricep to rescue age‐related skeletal muscle defects in mice.

**Results:**

mRNA levels of CFTR in human vastus lateralis exhibited significantly negative correlations with age (*r* = −0.87 in males and −0.62 in females, *p* < 0.05). Gastrocnemius mRNA level of CFTR decreased by 77.7 ± 4.6% in 29‐month‐old wild‐type mice compared to the 4‐month‐old. At 18‐month‐old, DF508 mice showed significantly reduced lean mass (by 35.6%), lower specific twitch force of the gastrocnemius (by 46.2%), decrease in fast/slow‐twitch muscle isoform ratio as well as downregulation of myogenic (e.g., MYOD and MYOG) or autophagy/mitophagy (e.g., LC3‐*β*) genes, compared to age‐matched wild‐types. Post‐injury gastrocnemius regeneration was found impaired in DF508 mice. Myoblast cultures from DF508 mice showed defective myogenic differentiation, which was reversed by overexpressing LC3‐β. In aged (> 15‐month‐old) mice, overexpressing CFTR or VX809 restored the expression of autophagy or myogenic genes, increased mitochondrial LC3‐β level and improved skeletal muscle mass and function.

**Conclusion:**

Age‐related reduction in skeletal muscle expression of CFTR impairs autophagy and myogenesis, exacerbating skeletal muscle aging. Enhancing CFTR might be a potential treatment strategy for age‐related skeletal muscle disorders.

## 1 Introduction

Skeletal muscles can be regenerated through the activation of adult myogenic progenitors for their differentiation into myoblasts and subsequent maturation into multinuclear muscle fibres [1]. Such adult myogenesis process is initiated upon physical injury or other stress/damage to skeletal muscles and complexed with sequential activation of myogenic genes as well as cellular/subcellular organisational changes [2]. The regenerative capacity of skeletal muscles decreases with age contributing to age‐related decline in skeletal muscle mass and strength, which is defined as sarcopenia [3]. Interestingly, autophagy, a catabolic process to degrade damaged cytoplasmic components or organelles, is known to be crucial to tissue regeneration [4] and exhibits age‐related decline [5]. In skeletal muscles, where mitochondria are easily damaged out of the high energy demand, the clearance of dysfunctional mitochondria through autophagy, known as mitophagy, is believed to be essential to skeletal muscle homeostasis and adult myogenesis [5, 6]. Accumulated evidence has also suggested that maladaptive mitophagy may account for age‐related disorders [7, 8, 9], and promoting autophagy may be beneficial to sarcopenia [10, 11].

CFTR is a cAMP‐activated anion channel abundantly expressed in epithelial cells, mutations (e.g., DF508) of which result in cystic fibrosis (CF), a genetic disease commonly seen in Caucasians [12]. Despite significant airway issues, CF patients often experience skeletal muscle weakness, among other complications [13]. CFTR knockout mice also showed skeletal muscle atrophy with disturbed Ca^2+^ activities [14, 15]. Although the expression of CFTR in the skeletal muscles was reported, whether CFTR plays a role in myogenesis for skeletal muscle homeostasis remained unclear. Interestingly, CFTR expression was reported to be age‐dependently downregulated in human sperm and prostates [16]. Moreover, CFTR deficiency has been associated with mitochondria dysfunction [17], autophagy problems [18], inflammation [19], oxidative stress [20], and cellular senescence in epithelial tissues [21]. We hypothesised that CFTR might play a role in skeletal muscle regeneration in aging. In the present study, we examined CFTR expression in skeletal muscles in humans and mouse models over different ages, explored its possible involvement in skeletal muscle myogenesis and autophagy in aging, and examined the effect of enhancing CFTR on skeletal muscles in aged mice.

## 2 Materials and Methods

### 2.1 Human Subjects

The vastus lateralis biopsies without obvious rupture, edema, or hematoma were collected at around 10 cm away from the surgery approaching site in fractured patients (9 male and 13 female) in the First Affiliated Hospital of Shantou University (Table S1). The uninjured muscle without or All procedures were approved by the Ethics Committee of the First Affiliated Hospital of Shantou University (B‐2023‐053). The informed consent was obtained from each human subject.

### 2.2 Animals

Wild‐type C57BL/6 and other mice including the senescence accelerated mouse P8 (SAMP8) and senescence accelerated mouse resistant 1 (SAMR1) were maintained at Laboratory Animal Services Centre, the Chinese University of Hong Kong or Centralized Animal Facilities of the Polytechnic University of Hong Kong. The CFTR mutant (DF508) transgenic mouse model (*Cftr* ^*tm1Kth*^) was imported as we previously documented [22, 23]. All procedures were approved by the Animal Ethics Committee of the Chinese University of Hong Kong (No.18‐215‐MIS) and Animal Subjects Ethics Sub‐committee of The Hong Kong Polytechnic University (No. 20‐21/259‐BME‐R‐HMRF). For general anaesthesia, mice were intraperitoneally injected with ketamine (100 mg per kg body weight) and xylazine (10 mg per kg body weight). To induce muscle injury, 15‐month‐old wild‐type and DF508 male mice were used. Under anaesthesia, 30 μL BaCl_2_ (1.2% in saline) was intramuscularly injected into the tibialis anterior muscle of the right limb of the mice. Female or male aged (29‐month‐old) wild‐type mice were used for local injection of VX809 (Selleckchem, S1565). In each mouse, 50 μL saline with VX809 (Selleckchem, S1565, 200 μM) or DMSO (0.2% v/v) as the vehicle control was locally injected into the midshaft of right femoris quadricep every two days for a total of 2 to 6 weeks. To overexpress CFTR in vivo, 15‐month‐old male or female mice were used. Adenovirus‐packaged CFTR (Adv‐CFTR, pAdeno‐MCMV‐CFTR‐T2A‐3Flag‐T2AmCherry) or null sequence (Adv‐vector, pAdeno‐MCMV‐3Flag‐T2A‐mCherry) purchased from OBiO Technology (Shanghai) was used. Under anaesthesia, 30μL saline containing 1.5 x 10^8^ PFU Adv‐CFTR or Adv‐vector was locally injected into the right gastrocnemius of each mouse.

### 2.3 Dual‐Energy X‐Ray Absorptiometry

Under anaesthesia, dual‐energy X‐ray absorptiometry (DEXA) (UltraFocus DXA, Faxitron, AZ, USA) was used to determine the whole‐body tissue composition. BiopticsVision software was used to define the region of interest (ROI) for the analysis of tissue compositions.

### 2.4 Ex Vivo Muscle Functional Test

An ex vivo muscle functional test system (800A, Aurora Scientific Inc., Newmarket, Canada) was used as previously reported [24]. Under anaesthesia, the gastrocnemii were collected with two‐point fixation at Achilles tendon and femur condylar. The fresh tissues were rinsed and stabilised for 15 min in Ringer solution (121 mmol/L NaCl, 5.4 mmol/L KCl, 1.2 mmol/L MgSO_4_.7H_2_O, 25 mmol/L NaHCO_3_, 5 mmol/L HEPEs, 11.5 mmol/L Glucose, 2.5 mmol/L CaCl_2_) gassed with 95% O_2_ and 5% CO_2_. The muscle was then electrically stimulated (1A, 300 ms, 150 Hz) twice to evoke tetanic contractions with a 5 min interval. The optimal length (L_0_) was confirmed when the new responsive force equalled the previous one after eliciting isometric twitch. The twitch force (F_0_) output was determined by the average of three twitch responses to a single stimulus (300 mA, 150 Hz) with a 1 min interval at the optimal length. The tetanic force (F_t_) was measured by three repeated continuous stimulations of 150 Hz for 300 ms with 5 min intervals. To evaluate the fatigability, the force after 30 consecutive pulses (300 ms tetanic stimuli at 150 Hz with 5 s intervals) was measured. The first and last recorded forces were used to calculate the force loss percentage. The wet muscle was weighed after functional measurements. Dynamic Muscle Control system (DMC v5.4 and 3.2; Aurora Scientific, Inc.) was used to record and analyse the data. The muscle cross‐sectional area (MCSA) was determined by muscle mass divided by L_0_ and density of mammalian skeletal muscle (1.06 mg/mm^3^). The specific twitch force (sF_0_) and specific tetanic force (sF_t_) were normalised by the MCSA (see supporting information).

### 2.5 Cell Culture

Primary myoblasts were isolated as previously reported [25]. Briefly, skeletal muscle tissues from mouse hindlimb were minced in Hank's Balanced Salt Solution (Invitrogen) and digested in 0.2% collagenase‐type A (Sigma) for 60 min and in 0.05% Trypsin (Invitrogen) for another 10 min at 37°C. A cell strainer (BD Biosciences, CA, USA) was used to filter out individual/single cells, which were then seeded on Matrigel (Corning, 356234) coated plates in DMEM/F12 (Gibco) supplemented with 20% fetal bovine serum (FBS), 10% horse serum (Invitrogen), 0.5% chick embryo extract (USbio, USA), 2.5 ng/mL bFGF (Gibco) and 100 U/mL penicillin and streptomycin (P/S, Gibco). Three days later, cells were re‐suspended and subjected to a pre‐plating protocol for five times. The unattached cells were used as primary myoblasts and cultured in Matrigel‐coated supports in the above‐mentioned growth medium. A mouse myoblast cell line, C2C12, was purchased from the American Type Culture Collection and maintained in DMEM (Gibco) containing 10% FBS and 100 U/mL P/S. To induce in vitro myogenic differentiation into myotubes, a high‐glucose DMEM (Gibco) with 2% horse serum was used and replenished every two days.

### 2.6 Western Blot

To extract proteins, tissues and cells were lysed in RIPA lysis buffer with complete mini protease/phosphatase inhibitor cocktail (Cell Signalling Technology, USA) followed with centrifugation at 15 000 rpm for 30 min at 4°C. Antibodies with 1:1000 dilution used were as follows: anti‐CFTR (Abcam, ab2784), anti‐MYOD (Abcam, ab64159), anti‐MYOG (Abcam, ab103924), anti‐PAX7 (Abcam, ab199010), anti‐LC3‐β (CST, 2775), anti‐COX IV (Abclonal, A6564) and anti‐GAPDH (Invitrogen, AM4300). Results were visualised on the GeneGnome XRQ (Syngene, Cambridge, UK). The mitochondrial and cytosol fractions were separated based on a modified common protocol [26].

### 2.7 Gene Knockdown and Overexpression

CFTR Stealth RNAi siRNAs (Catalogue number 1320001: MSS202937, MSS202939) or control StealthRNAi siRNAs (Invitrogen 2935300) (all obtained from Thermo Fisher Scientific) with lipofectamine 3000 (Invitrogen L3000075) was used for transfection of cells at 80% confluence to knockdown genes. To overexpress LC3‐β, a plasmid (addgene, 11546) was used. 1 μg plasmids with 1 μL lipofectamine was used to transfect 10 000 cells.

### 2.8 Statistics

Data were described as the mean ±S.D. Differences in measured variables between two groups were compared using Student's t‐test. For comparisons between more than two groups, One‐way analysis of variance (ANOVA) was used for single‐factor analysis, and Two‐way ANOVA was used for two‐factor analysis followed by Bonferroni's Multiple Comparison Test. Spearman's test was used for correlation analysis. Statistical significance was considered when *p* < 0.05.

Other methods can be found in supplementary information.

## 3 Results

### 3.1 Age‐Dependent Downregulation of CFTR in Skeletal Muscles

**FIGURE 1:**
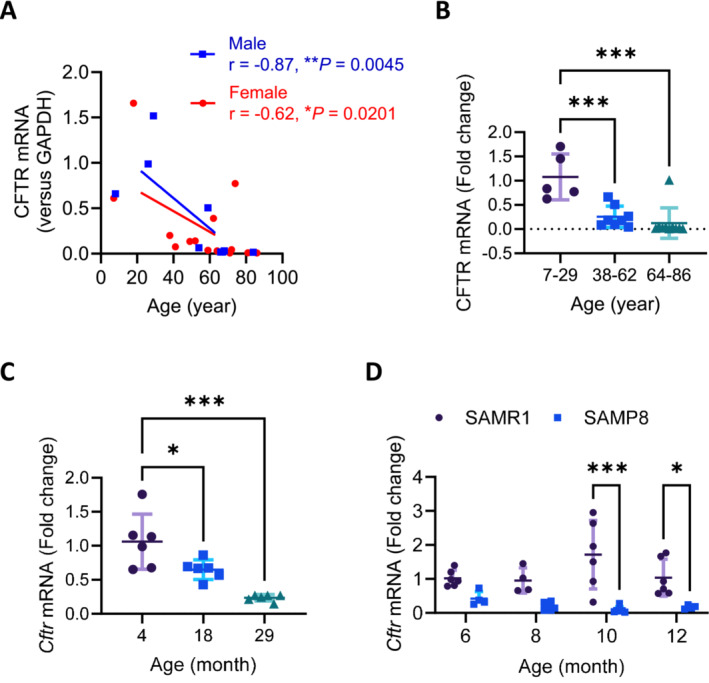
Age‐dependent downregulation of CFTR in human and mouse skeletal muscles. (A, B) Quantitative PCR (qPCR) analysis of CFTR mRNA levels in uninjured vastus lateralis biopsy samples from fractured patients (7 to 86 years old, 9 males and 14 females) in correlation with their ages (A) and comparison among different age groups (B). *p < 0.05, **p < 0.01, ***p < 0.01, Spearman correlation test in A and One‐way ANOVA with Bonferroni's multiple comparison test in B. (C, D) qPCR analysis of CFTR mRNA levels in gastrocnemii from wild‐type mice (C) and SAMP8 (a mouse model of accelerated senescence) mice in comparison with the SAMR1 control mice (D). * *p* < 0.05, *** *p* < 0.001, One‐way (C) and Two‐way (D) ANOVA with Bonferroni's multiple comparison test. *n* = 4 or 6.

We first asked if CFTR expression in skeletal muscles could be age‐dependently altered similarly as reported in other organ systems [16, 27]. We collected uninjured vastus lateralis biopsies from human subjects aged from 7 to 86 years old, which showed age‐dependent downregulation of CFTR at mRNA level in these human skeletal muscle tissues (Figure 1A,B). Next in mouse gastrocnemius tissues, mRNA level of CFTR was found to be age‐dependent declined (38.7% reduction from 4 to 18‐month‐old and 77.8% from 4 to 29‐month‐old respectively, Figure 1C). In addition, in SAMP8 mice [24], a mouse model of aging, CFTR mRNA levels in gastrocnemii were also significantly lower compared to the senescence‐resistant control mice (SAMR1) starting from 6 to 12‐month‐old (Figure 1D). These results suggest that CFTR expression in skeletal muscles decreases as age advances.

### 3.2 Age‐Dependent Skeletal Muscle Defects in CFTR‐Deficient Mice

**FIGURE 2:**
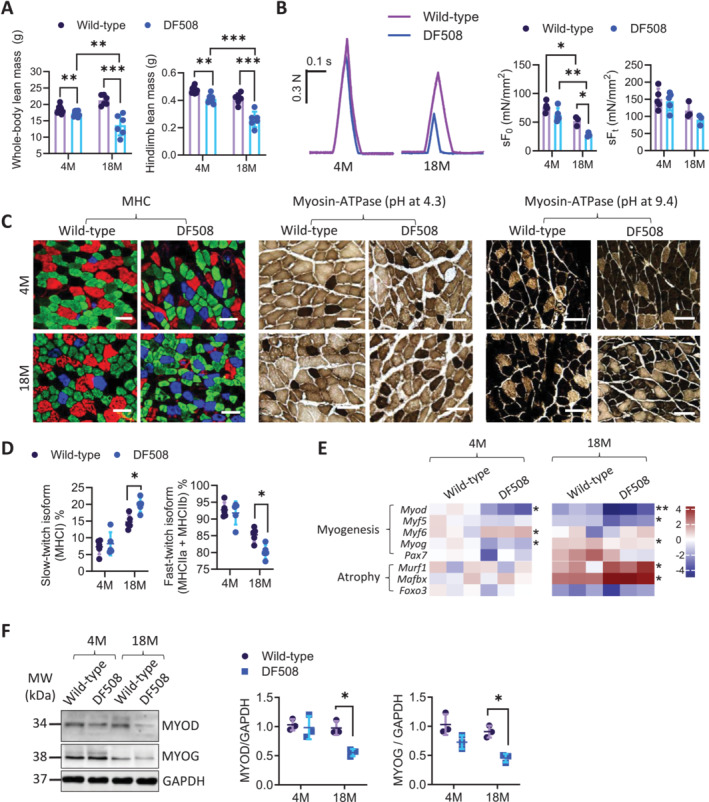
Age‐dependent skeletal muscle defects in CFTR‐deficient mice. (A) DEXAanalysis of whole‐body or lower hindlimb lean mass in male wild‐type or DF508 mice at 4‐ and 18‐month‐old (4 M and 18 M). **p < 0.01, ***p < 0.001, Two‐way ANOVA with Bonferroni's multiple comparison test. *n* = 6–10. (B) Ex vivo muscle functional test of gastrocnemii from male wild‐type or DF508 mice. *Left:* representative myographs of twitch forces. *Right:* quantification of specific tetanic force (sF_t_) and specific twitch force (sF_0_). *p < 0.05, **p < 0.01, Two‐way ANOVA with Bonferroni's multiple comparison test. *n* = 3. (C, D) Representative images (C) of MHC and myosin ATPase staining in gastrocnemii from male wild‐type or DF508 mice with quantification of slow‐ or fast‐ twitch fibres (D). *p < 0.05, Two‐way ANOVA with Bonferroni's multiple comparison test. *n* = 5. Scale bars, 20 μm. (E) Heatmap for mRNA levels (by qPCR) of myogenesis‐ and atrophy‐related genes in gastrocnemii from male wild‐type or DF508 mice. *p < 0.05, **p < 0.01, Two‐way ANOVA with Bonferroni's multiple comparison test. *n* = 3. (F) Western blots for MYOD or MYOG in gastrocnemii from male wild‐type and DF508 mice with quantification. *p < 0.05, **p < 0.01, Two‐way ANOVA with Bonferroni's multiple comparison test. *n* = 3.

To explore possible roles of CFTR in skeletal muscle aging, we examined the body mass of mice carrying CFTR loss‐of‐function mutation, DF508, at different ages. At 4‐month‐old, the lean mass (whole‐body or lower hindlimb) was about 6% lower (by DEXA) in DF508 mice than that in the wild‐type littermates (Figure 2A, Figure S1A,B). When the mice grew older to about 18‐month‐old, such a lean mass difference between the two groups became substantial, which was about 35% significantly lower in DF508 mice compared to the wild‐types (Figure 2A, Figure S1A,B). Measuring the wet weight of isolated gastrocnemii confirmed the significant difference between the DF508 and wild‐type at 18‐month‐old (S1C). We then conducted functional tests of skeletal muscles from the mice by measuring the contractions of freshly harvested gastrocnemius in response to electrical stimuli ex vivo (see methods). As shown in Figure 2B, the specific twitch force (sF_0_) of the gastrocnemius decreased from 4 to 18‐month‐old in both groups. At 18‐month‐old, DF508 gastrocnemii had significantly lower sF_0_ (28.09 ± 3.59 mN/mm^2^) compared to the wild‐types (52.16 ± 8.02 mN/mm^2^). The tetanic force (sF_t_, Figure 2B) was slightly weaker in DF508 compared to the wild‐type at both ages, though with no statistical significance. We further did histological analysis of muscle types and plasticity by staining for myosin heavy chain (MHC)/myosin ATPase (Figure 2C,D) and laminin (Figure S1D), which showed that at 18‐month‐old, DF508 mice had a significant increase in slow‐twitch isoform and a decrease in fast‐twitch isoform, as well as a decreased cross‐sectional area (CSA) of myofibers (Figure S1D) in the gastrocnemii compared to the wild‐types. Such differences between DF508 and wild‐type groups were all not evident at 4‐month‐old. We also examined neuromuscular junction transduction in DF508 mice at 20‐month‐old, which showed that although triceps surae contractile responses to sciatic nerve stimulations were similar between DF508 and wild‐type, the area, branching and discontinuity of post‐synaptic junctions in the extensor digitorum longus (as indicated by AChR labelling, see methods) were increased in DF508 compared to the wild‐types (Figure S2). These results together suggest that CFTR deficiency can exacerbate age‐dependent declines in skeletal muscle mass and function.

### 3.3 Impaired Myogenesis by CFTR Deficiency

**FIGURE 3:**
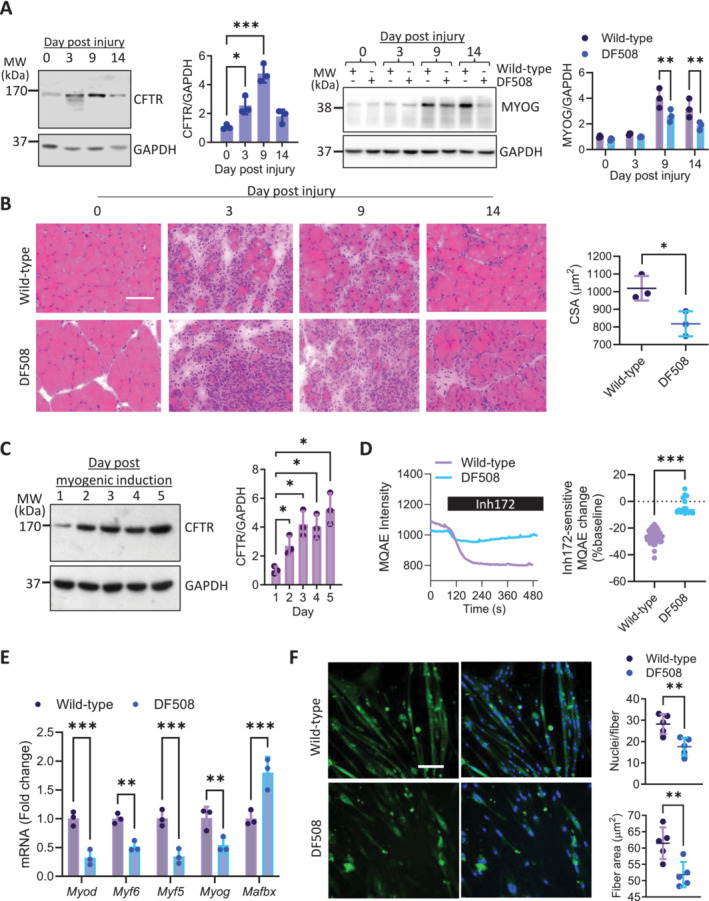
Impaired myogenesis by CFTR deficiency. (A, B) Western blots with quantification for CFTR or MYOG (A) and H&E staining with quantification of cross‐sectional area (CSA) (B) in tibialis anterior from male wild‐type or DF508 mice (18‐month‐old) collected at day 0, 3, 9 and 14 post injury (see methods). *p < 0.05, **p < 0.01, ***p < 0.001, One‐way or Two‐way ANOVA with Bonferroni's multiple comparison test in A and unpaired t‐test in B. *n* = 3. GAPDH was used as a loading control. (C) Western blots with quantification for CFTR in mouse primary cultured myoblasts (PMBs) at day 1 to 5 after myogenic differentiation was induced in vitro (see methods). *p < 0.05, One‐way ANOVA with Bonferroni's multiple comparison test. *n* = 3. GAPDH was used as a loading control. (D) Real‐time measurement of intracellular Cl^−^ level (inversely correlated with MQAE intensity, see methods) of wild‐type or DF508 PMBs when a CFTR inhibitor, inh‐172 (10 μM), was added into the bath. Inh172‐sensitive MQAE change was quantified. ***p < 0.001, unpaired t‐test. *n* = 17–43. (E, F) qPCR for myogenesis or atrophy related genes and immunofluorescence staining for MHC IIa in wild‐type or DF508 PMBs at day 3 (E) or day 5 (F) post the myogenetic differentiation induction. **p < 0.01, ***p < 0.001, unpaired t‐test. *n* = 3. DAPI was used to label nuclei, which were counted. MHC IIa‐positive area was quantified as fibre area. Scale bar, 50 μm **p < 0.01, ***p < 0.001, unpaired t‐test. *n* = 5.

To understand the age‐dependent skeletal muscle defects observed in CFTR deficient mice, we examined the transcription of genes key to myogenesis or muscle atrophy in the mouse gastrocnemii. Myogenic genes *Myod* and *Myog* were significantly down‐regulated and primary regulators of muscle atrophy (*Murf1*, and *Mafbx*) were upregulated in DF508 mice compared to the wild‐types at 18‐month‐old (Figure 2E). Western blot analysis confirmed that gastrocnemii from DF508 mice had age‐dependent (from 4 to 18‐month‐old) decrease in MYOD and MYOG protein expression to a more severe extent compared to the wild‐types (Figure 2F), suggesting that CFTR deficiency might accelerate the impairment of myogenesis in aging. We next tested the possible involvement of CFTR in myogenesis using a mouse model of skeletal muscle injury and regeneration (see methods). As shown in Figure 3A, protein expression of CFTR in gastrocnemii was found to be robustly increased post the injury starting from day 3 (by 2.55‐fold) and peaked at day 9 (by 4.78‐fold) in wild‐type mice. Interestingly, MYOG protein level in gastrocnemii was found elevated only on day 9 and sustained on day 14 post the injury, which was later than the observed CFTR upregulation on day 3. Importantly, DF508 mice showed much lower levels of MYOG on day 9 and 14, as compared to the wild‐types. Consistently, histological analysis revealed that the regenerated myofibers with centre‐localised nuclei markedly accumulated in wild‐type mice from day 9 post‐injury, while such regenerative capacity was absent in DF508 mice (Figure 3B). The CSA of muscle fibres on day 14 post‐injury, indicating regeneration outcomes, were found significantly smaller in DF508 mice than that in the wild‐type mice.

Given such impaired post‐injury myogenesis observed in DF508 mice, we asked whether CFTR plays a direct role in myogenesis. We first counted the number of PAX7‐postive cells (satellite cells) in the mice. Although both wild‐type and DF508 mice had age‐related reduction in PAX‐7 positive cells from 4 to 18‐month‐old, there was no significant difference between the two groups at both ages (Figure S1D). We then used myoblasts cultures including mouse primary myoblasts (PMBs, Figure S3A) and C2C12, a cell line of mouse myoblast, to examine the role of CFTR in myogenesis. We confirmed CFTR expression at both mRNA and protein levels in the PMBs and C2C12 (Figure S3B,C). In addition, we used MQAE (a Cl^−^ sensitive fluorescence dye) measurement of intracellular level of Cl^−^ to examine possible Cl^−^ channel function of CFTR in these myoblasts. A selective CFTR inhibitor (Inh172, 10 μM) induced intracellular Cl^−^ increase (indicated by MQAE intensity drop) in wild‐type PMBs, which were largely diminished in cells from DF508 mice, suggesting functional expression of CFTR in the PMBs to mediate Cl^−^ efflux (Figure 3D). Interestingly, after myogenic differentiation was induced in PMB cultures (see methods), the expression of CFTR (at both mRNA and protein levels) was gradually increased (Figure 3C, Figure S3D), similarly as seen in vivo myogenesis (Figure 3A). Examining these PMBs on day 5 post myogenic treatment showed that DF508 PMBs had significantly lower mRNA expression levels of myogenic genes *Myod*, *Myf5*, *Myf6* and *Myog*, but higher *Mafbx*, a muscle atrophy marker (Figure 3E). Counting the muscle fibres in these cultures on day 5 post myogenic treatment showed a reduced number of differentiated muscle fibres in DF508 PMBs compared to wild‐type ones (Figure 3F). Moreover, PMBs from DF508 mice contained less MYOD‐positive cells (Figure S1E) and exhibited lower capacity of Ca^2+^ mobilisation than wild‐type cells (Figure S3F,G). Consistent results were achieved in PMBs or C2C12 cells treated with siRNA‐based CFTR knockdown (Figure S4A–F), which together suggest impaired myogenic capacity of myoblasts with CFTR deficiency.

### 3.4 CFTR‐Deficiency Exacerbates age‐Related Decline of Autophagy/Mitophagy in Skeletal Muscles

**FIGURE 4:**
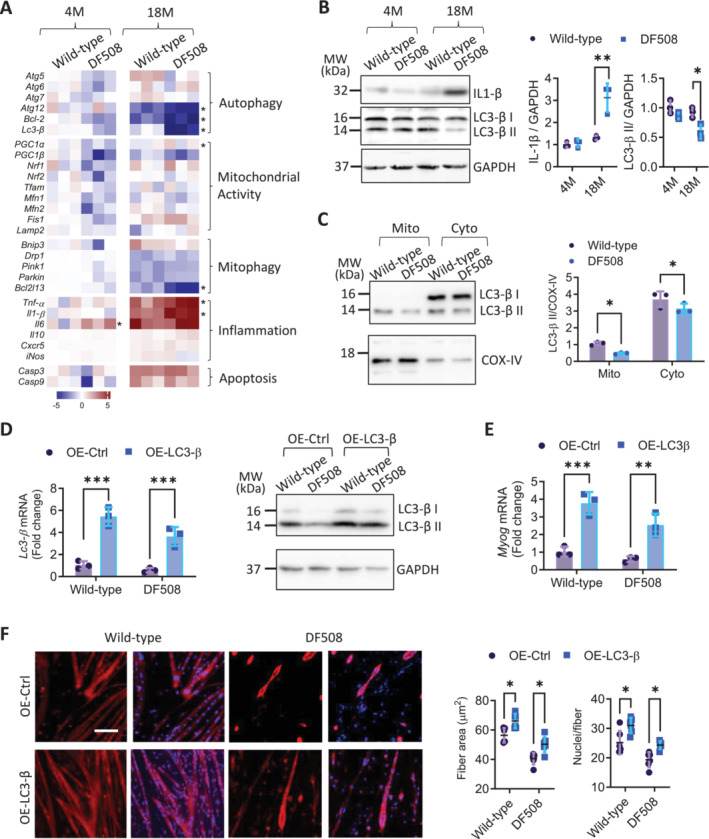
CFTR‐deficiency exacerbates age‐related decline of autophagy/mitophagy in skeletal muscles. (A, B) Heatmap for mRNA levels (by qPCR, A) of indicated genes and western blots (B) for IL1‐*β* and LC3‐*β* in gastrocnemii collected from male wild‐type or DF508 mice at 4‐ or 18‐month‐old. *p < 0.05, **p < 0.01, Two‐way ANOVA with Bonferroni's multiple comparison test. *n* = 3. (C) Western blots for LC3‐*β* in mitochondrial (mito) and cytosol (cyto) fractions of the gastrocnemii from wild‐type or DF508 mice (male, 18‐month‐old). *p < 0.05, Two‐way ANOVA with Bonferroni's multiple comparison test *n* = 3. COX‐IV was probed as a loading control. (D, F) qPCR and western blotting for LC3‐*β* (D), qPCR for MYOG (E), and immunofluorescence staining for MHC IIa (F) in wild‐type or DF508 PMBs with LC3‐*β* overexpression (OE‐LC3‐*β*) or treated with vector plasmids as the control (OE‐Ctrl) at day 3 (D and E) or day 5 (F) post myogenic differentiation induction. DAPI was used to label nuclei, which were counted. MHC IIa‐positive area was quantified as fibre area. Scale bar, 50 μm. **p < 0.01, ***p < 0.001, Two‐way ANOVA with Bonferroni's multiple comparison test. *n* = 3.

We next asked how CFTR‐deficiency impaired the myogenesis. We examined gastrocnemius tissues isolated from wild‐type and DF508 mice for expression of genes related to mitochondrial function, autophagy (mitophagy), inflammation, and apoptosis, which interestingly revealed that, in addition to the surge of inflammatory factors *Tnf‐a* and *Il‐1β*, autophagy related genes including *Atg12*, *Bcl‐2*, *Lc3‐β*, and *Bcl2l13* related to mitophagy were significantly inhibited in DF508 mice compared to the paired wild‐types at 18‐month‐old (Figure 4A). Transmission electron microscopy (TEM) analysis showed that autophagosome and mitophagosome numbers were age‐dependently decreased from 4‐ to 18‐month‐old in both wild‐type and DF508 gastrocnemii, which, however, was significantly exacerbated in DF508 mice compared to the wild‐types (Figure S5). Western blotting for the autophagy mediator, LC3‐β, showed that the level of the autophagy‐activated form of LC3‐β, LC3‐β II, was significantly reduced together with the elevation in IL1‐β level in gastrocnemii from 18‐month‐old DF508 mice, as compared to the wild‐types (Figure 4B). We further conducted protein fractionation and found that mitochondrial LC3‐β II level was significantly reduced by 39.6% in DF508 gastrocnemii compared to that of the wild‐types (Figure 4C), suggesting defective mitophagy in the DF508. Consistently, the downregulation of LC3‐β was also detected in PMBs with siRNA‐based CFTR knockdown (Figure S4D). To test if the CFTR‐deficiency‐induced LC3‐β downregulation would underlie the observed myogenic defects, we over‐expressed LC3‐*β* in PMBs (Figure 4D), which, indeed, increased MYOG mRNA level (Figure 4E) and reversed the impaired myotube formation (Figure 4F) in DF508 PMBs.

### 3.5 Effect of CFTR Overexpression on Skeletal Muscles in Aged Mice

**FIGURE 5:**
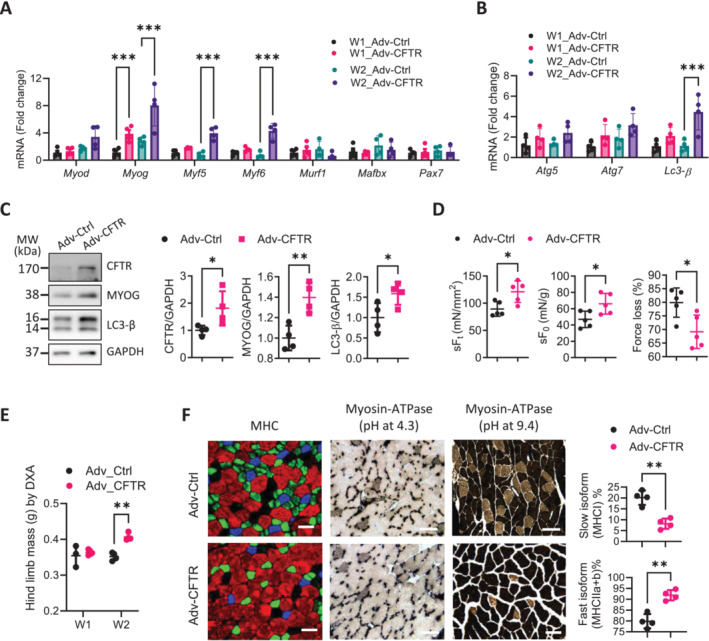
CFTR overexpression promotes skeletal muscle function in aged mice. (A, B) qPCR for myogenesis/atrophy‐ (A) and autophagy‐ (B) related genes in gastrocnemii collected from 15‐month‐old female wild‐type mice at week 1 (W1) or week 2 (W2) after injected with adenoviruses containing human CFTR gene (Adv‐CFTR) or control viruses (Adv‐Ctrl). ***p < 0.001, Two‐way ANOVA with Bonferroni's multiple comparison test *n* = 4. (C, D) Western blot for CFTR, MYOG, and LC3‐*β* (C), and muscle functional tests of sF_t_, sF_0_ and force loss (D, see methods) in gastrocnemii collected at W2. *p < 0.05, **p < 0.01, unpaired t‐test *n* = 4 (C) and 5 (D). (E) DEXA measurement of lean mass of the lower hindlimb injected with Adv‐CFTR or Adv‐Ctrl at W1 or W2. **p < 0.01, Two‐way ANOVA with Bonferroni's multiple comparison test *n* = 3–4. (F) Representative images of MHC and myosin ATPase staining with quantification of slow‐ or fast‐ twitch fibres in the Adv‐CFTR‐ or Adv‐Ctrl‐treated gastrocnemii collected at W2. **p < 0.01, unpaired t‐test *n* = 4. Scale bars, 20 μm.

The above results suggested that CFTR deficiency in aged skeletal muscles impaired autophagy and myogenesis. We next tested the effect of overexpressing CFTR. Adenoviruses conjugated with human CFTR gene (Adv‐CFTR) were locally injected into the gastrocnemius in 15‐month‐old mice (See methods). The expression of the exogeneous CFTR was detected in the lower hindlimb at 1 and 2 weeks after the virus injection (Figure 5C and Figure S6A). At week 1, the mRNA level of a myogenic gene, *Myog*, was found significantly elevated in the gastrocnemii treated with Adv‐CFTR as compared to that of control adenovirus (Adv‐Ctrl)‐treated ones. At week 2, other myogenetic genes, *Myog*, *Myf5* and *Myf6*, were all significantly upregulated by Adv‐CFTR (Figure 5A), suggesting enhanced myogenic capacity by the treatment. We also tested the expression of autophagy genes (*Atg 5*, *Atg7* and *Lc3‐β*), which revealed that *Lc3‐β* mRNA level was significantly upregulated (by 4.55‐fold) at week 2 in Adv‐CFTR treated gastrocnemii (Figure 5B). Western blotting confirmed that together with CFTR overexpression, the protein levels of MYOG and LC3‐*β* were higher in Adv‐CFTR injected gastrocnemii at week 2 (Figure 5C). Importantly, the Adv‐CFTR treatment for 2 weeks increased the contractility (Figure 5D), mass (Figure 5E, Figure S6B), fast/slow‐twitch muscle isoform ratio (Figure 5F), and myofiber CSA (Figure S6C) of the treated gastrocnemii, compared to the Adv‐Ctrl treated ones, suggesting the potential of enhancing CFTR in rescuing skeletal muscle disorders.

### 3.6 Effect of CFTR Modulator on Skeletal Muscles in Aged Mice

**FIGURE 6:**
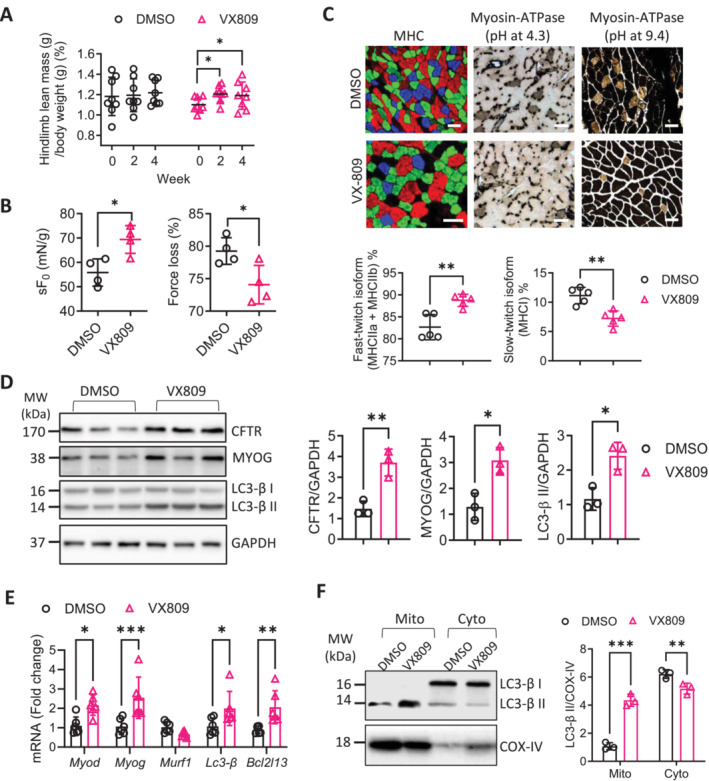
Effect of CFTR modulator (VX809) on skeletal muscles in aged mice (A) DEXA measurement of lean mass in the lower hindlimb of 29‐month‐old female wild type mice injected with VX809 or DMSO as control at week 0, 2, and 4 post the injection. Data are normalised by body weight. *p < 0.05, paired t‐test. *n* = 8. (B–E) Muscle functional tests of sF_0_ and force loss (B), MHC and myosin ATPase staining with quantification of slow‐ or fast‐ twitch fibres (C, scale bars, 20 μm), western blots for CFTR, MYOG and LC3‐*β* and qPCR for indicated genes in the gastrocnemii injected with DMSO or VX809 collected at week 4 post the injection. *p < 0.05, **p < 0.01, ***p < 0.001, unpaired t‐test *n* = 3–5. (F) Western blots for LC3‐*β* with quantification in mitochondrial (mito) and cytosol (cyto) fractions of the VX809‐ or DMSO‐treated gastrocnemii collected at week 4 post the injection. COX‐IV was used as a loading control. **p < 0.01, ***p < 0.001, Two‐way ANOVA with Bonferroni's multiple comparison test. *n* = 3.

We next tested the possible effect of VX809, a CFTR modulator that enhances CFTR, in rescuing age‐related skeletal muscle defects. We performed in vitro tests first, which revealed that VX809 (100 nM, 72 h) up‐regulated *Myf5* (by 5.49‐fold) and *Lc3‐β* (by 4.42‐fold) (Figure S7A,B) and promoted the myofiber formation (Figure S7C). We continued to test VX809's effect in vivo and performed local injection into the femoris quadriceps in aged (29‐month‐old) mice (see methods). Results showed that the VX809 treatment for 2 to 4 weeks increased hindlimb mass compared to that before the treatment (week 0) (Figure 6A and Figure S8A–C). At week 4, functional test showed improvement in sF_0_ and reduction in force‐loss by VX809 treatment, compared to the control group treated with the vehicle, DMSO (Figure 6B). Morphological analysis revealed that myofiber CSA (Figure S8D) and proportion of fast‐twitch muscle fibre were increased by VX809 (Figure 6C and Figure S8E). Examining myogenesis or autophagy genes in the treated muscles showed that CFTR, MYOD, MYOG, LC3‐*β* and BCL2L13 were upregulated compared to the DMSO‐treated controls at 4 weeks post treatment (Figure 6D,E, and Figure S8F). Importantly, both overall (Figure 6D) and mitochondrial (Figure 6F) LC3‐*β* II levels were substantially increased (by 4‐fold) after 4 weeks of VX‐809 treatment (Figure 6F). TEM analysis confirmed that autophagosome and mitophagosome areas were increased, and the impaired mitochondria area was reduced in the VX‐809 treated gastrocnemii, as compared to the DMSO controls (Figure S8G). These results suggest that CFTR enhancement by VX‐809 boosted the autophagy and myogenic capacities, improving muscle mass and function in aged mice.

## 4 Discussion

In summary, the present study has demonstrated that the expression of CFTR in human and rodent skeletal muscles exhibits significant age‐dependent decline. Deficiency in CFTR impairs skeletal muscle autophagy and myogenesis, exacerbating skeletal muscle aging. Overexpression of CFTR or pharmaceuticals that enhance CFTR restored autophagy and myogenic capacities, and improved skeletal muscle functions in aged mice. CFTR is therefore revealed as a novel target for treating age‐related skeletal muscle disorders.

Skeletal muscle weaknesses had been noted in CF patients and largely attributed to their physical inactivity or nutrition imbalance [28, 29, 30]. The present study has shown, to the best of our knowledge, for the first time that CFTR plays a direct role in skeletal myogenesis. First, mRNA and protein expression of CFTR were detected and verified in human and mouse skeletal muscle tissues, isolated mouse myoblasts and a mouse myoblast line, C2C12. Its channel activity to mediate Cl^−^ efflux was also detected in primary myoblasts. In addition, CFTR expression was found upregulated during the induced myogenetic differentiation in vitro as well as in skeletal muscle regeneration post injury in vivo. Second, skeletal muscles tissues from DF508 mice showed downregulation of myogenic genes together with functional defects especially at an older age. Third and importantly, DF508 mice exhibited impaired skeletal muscle regeneration post injury; primary myoblasts isolated from DF508 mice or myoblasts treated with siRNA‐based CFTR knockdown both showed defective myogenic differentiation in vitro, indicating the direct and significant involvement of myoblast CFTR in skeletal myogenesis. Moreover, overexpressing CFTR in vivo promoted myogenic genes activation and improved skeletal muscle functions, although CFTR may also be overexpressed in differentiated myotubes/fibres to exert the effect in the model. Of note, previous studies showed that the loss of CFTR exaggerated muscle atrophy [14, 15]. It should be noted that although atrophy marker genes were high in skeletal muscles of aged DF508 mice, in vivo overexpression of CFTR did not change atrophy marker genes but upregulated myogenic genes significantly, suggesting a main role of CFTR in promoting myogenesis to sustain skeletal muscle heath.

In the effort to understand how CFTR plays a role in myogenesis, we achieved data to suggest that CFTR regulates autophagy. Loss of CFTR exacerbated impaired mitochondrial autophagy in aging. In particular, the expression levels of CFTR and LC3‐*β* were consistently found to be in positive correlation in the gain and loss experiments both in vivo and in vitro. These findings support the involvement of CFTR in maintaining autophagy in myogenic cells, similarly as reported in epithelial cells [31]. Of note, our data support the functional expression of CFTR in plasma/surface membrane of myoblasts to mediate Cl^−^ efflux, which could influence membrane depolarization and thus Ca^2+^ as we demonstrated in other excitable cells [22, 32]. Indeed, we observed that Ca^2+^ was impaired in DF508 myoblasts, consistent with previous studies [14]. CFTR‐deficiency‐induced disturbance in Ca^2+^ homeostasis may underlie the impaired autophagy. Additionally, CFTR mutation/deficiency is reported to associate with endoplasmic reticulum (ER) stress and pro‐inflammation. We also found Bcl2l13, a Bcl‐2 like and ER‐resident protein, as well as and pro‐inflammation genes to be significantly downregulated in aged DF508 skeletal muscles. Possibly, CFTR deficiency‐associated ER stress and inflammation may also underlie CFTR‐regulated autophagy/mitophagy, which awaits further investigation. Under most of the conditions in the present study, LC3‐*β* I is the dominant form (higher than LC3‐*β* II level). LC3‐*β* II became the dominant form only when 1) overexpression of LC3‐*β* was done in cells or 2) VX809 was applied in the tissues. Since LC3‐*β* II is an indicator for the activation of autophagy, it is reasonable that autophagy is more active when LC3‐*β* is overexpressed. VX809 strongly promoted LC3‐*β* II level, particularly the mitochondrial LC3‐*β* II level, suggesting its capacity in efficiently activating autophagy/mitophagy. The drug may be more effective than CFTR overexpression to promote autophagy/mitophagy. It should be noted that both mitochondrial and cytoplasmic LC3‐*β* II levels were found to be affected in DF508 condition, while the VX809 treatment in vivo enhanced the mitochondrial but not the cytoplasmic LC3‐*β* II level. This is probably because DF508 mutation causes the mutant CFTR protein to be stuck in ER, which is different than the condition of CFTR downregulation in aged animals where VX‐809 was applied. VX‐809 is noted to act on and enhance wild‐type CFTR [33]. Our results supported that VX‐809 treatment enhanced the mRNA and protein expression levels of the wild‐type CFTR. How exactly mitophagy is promoted by the VX809‐enhanced wild‐type CFTR awaits further study. Of note, in our hands, the overexpression to elevate LC3‐*β* by about 6‐fold and 2‐fold at mRNA and protein levels, respectively, promoted the myogenesis. While, higher doses of LC3‐*β* overexpression resulted in cell death in myoblast cultures, suggesting that overpromoted autophagy could be harmful too.

It should be noted that CFTR deficiency in skeletal muscles could be a problem beyond CF or CFTR mutation. As shown presently, the CFTR expression levels in skeletal muscles were found to be in a significant negative‐correlation with the ages in both humans and wild‐type mice. Such age‐dependent downregulation of CFTR could be a result of hormonal decline or cell stress as previously reported, although the exact cause awaits further investigation. The observed age‐dependent difference in skeletal muscles between wild‐type and DF508 mice, together with demonstrated role of CFTR in skeletal myogenesis and autophagy, has suggested CFTR deficiency or malfunction to contribute to skeletal muscle aging. It is therefore revealed CFTR to be a target for treating age‐related skeletal muscle disorders. Promising data achieved presently by local administration of either CFTR‐containing virus or VX809 in aged wild‐type mice have confirmed the potential of enhancing CFTR as a new therapeutic strategy for sarcopenia or other skeletal muscle disorders in a general population.

## Conflicts of Interest

The authors declare no conflicts of interests.

## Supporting information


**Table S1.** Information about the human subjects involved in the study, detailing their gender, age, and the reason for their surgery.
**Table S2.** List of primers used in the study, with ‘H’ indicating human genes and ‘M’ indicating mouse genes.
**Figure S1.** Age‐dependent skeletal muscle defects in CFTR mutant (DF508) mice.
**Figure S2.** Neuromuscular junction function test in DF508 mice.
**Figure S3.** Involvement of CFTR in myogenic differentiation in vitro.
**Figure S4.** Effect of CFTR knockdown on myogenic differentiation in vitro.
**Figure S5.** Mitochondria and autophagosome changes in DF508 mice.
**Figure S6.** Effect of adenovirus‐mediated overexpression of CFTR on skeletal muscles in aged mice.
**Figure S7.** Effect of VX809 treatment on myoblasts in vitro.
**Figure S8.** Effect of VX809 on skeletal muscles in aged mice.
