# Supplementary material for: Defective Cystic Fibrosis Transmembrane Conductance Regulator Accelerates Skeletal Muscle Aging by Impairing Autophagy/Myogenesis
Source: J Cachexia Sarcopenia Muscle. 2025 Jan 29;16(1):e13708. doi: 10.1002/jcsm.13708 (PMC11780116; doi:10.1002/jcsm.13708)
Supplement: Supplementary file 1 — Table S1. Information about the human subjects involved in the study, detailing their gender, age, and the reason for their surgery. Table S2. List of primers used in the study, with ‘H’ indicating human genes and ‘M’ indicating mouse genes. Figure S1. Age‐dependent skeletal muscle defects in CFTR mutant (DF508) mice. Figure S2. Neuromuscular junction function test in DF508 mice. Figure S3. Involvement of CFTR in myogenic differentiation in vitro. Figure S4. Effect of CFTR knockdown on myogenic differentiation in vitro. Figure S5. Mitochondria and autophagosome changes in DF508 mice. Figure S6. Effect of adenovirus‐mediated overexpression of CFTR on skeletal muscles in aged mice. Figure S7. Effect of VX809 treatment on myoblasts in vitro. Figure S8. Effect of VX809 on skeletal muscles in aged mice. [file JCSM-16-e13708-s001.pdf]

Supplementary information

Defective cystic fibrosis transmembrane conductance regulator accelerates skeletal muscle aging by impairing autophagy/myogenesis

Ziyi Chen[1,2], Jiankun Xu[1], Peijie Hu[2], Wanting Du[2], Junjiang Chen[2], Xiaotian Zhang[2], Wei Zhou[3], Jiayang Gao[4], Yuantao Zhang[1], Bingyang Dai[1], Guangshuai Nie[5], Jun Hu[5], Liangbin Zhou[1], Shunxiang Xu[1], Hisao Chang Chan[6], Wing-hoi Cheung[1], Ye Chun Ruan[2*], Ling Qin[1*].

[1] *Musculoskeletal Research Laboratory, Department of Orthopedics & Traumatology, The Chinese University of Hong Kong, Hong Kong SAR, China*

[2] *Department of Biomedical Engineering, Faculty of Engineering, The Hong Kong Polytechnic University, Hong Kong SAR, China*

[c] *State Key Laboratory of Respiratory Disease for Allergy Shenzhen Key Laboratory of Allergy & Immunology School of Medicine, Shenzhen University, Shenzhen, China*

[4] *School of Life Sciences, Centre for Cell & Developmental Biology and State Key Laboratory of Agrobiotechnology, The Chinese University of Hong Kong, Hong Kong SAR, China*

[5] *Orthopaedic Research Centre, Department of Orthopaedics, the First Affiliated Hospital of Shantou University Medical College, Shantou, China*

[6] *Epithelial Cell Biology Research Centre, School of Biomedical Sciences, Faculty of Medicine, The Chinese University of Hong Kong, Hong Kong SAR, China*

[*] Corresponding authors:

(1) Prof. Ye Chun Ruan, email: sharon.yc.ruan@polyu.edu.hk

(2) Prof. Ling Qin, email: lingqin@cuhk.edu.hk

Supplementary information

Other methods

Flow cytometry

The gastrocnemii were isolated from 14-month-old wild-type and DF508 mice and digested in 0.2% collagenase type A (Sigma) for 90 min and in 0.05% Trypsin (Invitrogen) for another 30 min at 37°C. A cell strainer (BD Biosciences, CA, USA) was used to filter out individual/single cells, and fixed in 4% PFA for 20min. The cells were permeabilized in 0.2% Triton X-100 and blocked by 4% Bovine Serum Albumin (Sigma). Next, incubate in dilution buffer (2% FBS in DMEM) with anti-MYOD (Abcam, ab64159, 1:100) and secondary antibody Donkey anti-Rabbit IgG 488 (Invitrogen, A-21206, 1:500). Unstained cells were used as a negative control. The samples were scanned and analyzed by BD FACSVia Flow Cytometer and BD Accuri C9 software.

Ex vivo NMJ functional test

The NMJ function using the nerve-muscle complex (sciatic nerve-triceps surae) was tested by the same ex vivo system. Similarly, the optimal length (L0) of the muscle was measured when maximal force was generated by eliciting isometric twitch (300 mA, 0.2 ms pulse width). Under the L0, the muscle and nerve were electronically stimulated separately by a single stimulus with 1 min interval to calculate the F0 (Muscle stimulus: 300 mA, 0.2 ms pulse width; Nerve stimulus: 5 mA, 0.8 ms pulse width). Then, the muscle (300 mA, 300 ms duration, 0.2 ms pulse width, 50 Hz stimulation frequency) and nerve (5 mA, 300 ms duration, 0.8 ms pulse width, 50 Hz stimulation frequency) were activated by a tetanic stimulus with 2 min interval to measure the Ft, respectively. The fatigue capacity of muscle or nerve was evaluated by consecutive 100 cycles of F0 stimulation with a rest time of 0.7s. Neurotransmission failure (NF) and intra-tetanic fatigue (IF) of muscle and NMJ can be generated from the two separate consecutive tetanic stimuli.

$$MCSA\ (mm^2) = \frac{1000 \times MM\ (g)}{\left(\frac{L}{L_0}\right) \times D \times 10 \times L_0 (cm)}$$

$$SF_0 (mN/mm^2) = \frac{F_0\ (mN)}{MCSA\ (mm^2)}$$

$$SF_t (mN/mm^2) = \frac{F_t\ (mN)}{MCSA\ (mm^2)}$$

Histological analysis

Muscle samples treated by cold 2,2-Dimethylbutane were cryo-sectioned for histological examination. The tissues sectioned at 8 µm thick (Cryostar NX70, Thermo Scientific, MA, USA) were mounted on silane coated glass slides. Immunofluorescence (IF) staining of myosin heavy chain (MHC) was performed for muscle fibre typing based on previous protocol (2). Primary antibodies against MHCI (BA-F8), MHC IIa (SC-71), and MHC IIb (BF-F3) (DSHB, IA, USA) were mixed to form the primary antibody cocktail. Secondary antibodies including Alexa Fluor 350 IgG2b, Alexa Fluor 488 IgG1, and Alexa Fluor 555 IgM (Invitrogen, CA, USA) were diluted and mixed to form the secondary antibody cocktail. The concentration of

both cocktails was 4 μg/mL. Twenty images of positive staining were randomly acquired from the whole cross-sections of gastrocnemii and areas of MHC IIa (green), MHC IIb (red), and MHC I (blue) were quantified by Image J software. The sections were fixed by 4% paraformaldehyde (PFA) for IF staining of CFTR. The antigen retrieval was performed by boiling the sections on slides in EDTA alkaline buffer for 20 minutes. After 30 min blocking with 5% BSA, sections were probed by 1:100 anti-CFTR (Abcam, ab2784), 1:100 anti-PAX7 (Abcam, ab199010) and 1:200 anti-Laminin (Sigma, L0663) in a humidified chamber at 4°C overnight. The slides were washed three times with PBS and then incubated with 1:500 fluorescent-conjugated secondary antibody at room temperature (protected from light) for 60 minutes. At least ten scopes were randomly selected to quantify the cross-sectional area (CSA) outlined by Laminin by image J (3). Data analyses were blinded. The researchers performing the imaging acquisition and scoring were unaware of treatment condition given to sample groups analyzed.

The Myosin ATPase staining was performed based on reported protocol (4). The muscle sections were incubated in preincubating solution (0.143 mol/L barbital acetate, 0.1 N HCl, and deionized water) at pH at 4.6 for 5 minutes or pH at 9.4 for 10 minutes and then in ATPase solution (Sigma) at pH at 9.4 for 30 minutes at 37°C. The sections were incubated in 0.068 mol/L $CaCl_2$ and 0.084 mol/L $CoCl_2$ for 10 minutes, respectively, followed with washing in 1:20 sodium barbital (BDH Laboratory Supplies, Poole, England) and deionized water for five times. The sections were then immersed in 2% (*v/v*) ammonium sulfide solution (Sigma) for 10 to 20 seconds. After dehydration, the sections were mounted. Different muscle fibers were distinguishable by different color intensities. For sections incubated with pH at 4.6, the lightest colors representing Type IIA fibers, intermediate colors representing type IIB fibers, and the darkest colors representing type I fibers. For those with pH at 4.6, the lightest colors representing Type I fibers, intermediate colors representing type IIB fibers, and the darkest colors representing type IIA fibers. The fiber area of various types was quantified by Image J and combined with MHC staining.

For the staining of NMJ elements, 18-month-old mice with or without DF508 mutation were rested under inhalation anesthesia (5% isoflurane) and confirmed by deep tendon reflex. The extensor digitorum longus was isolated after PFA perfusion-fixed through the left ventricle. Then the samples were fixed at 4°C lasting 24 h for whole-mount muscle preparation. Muscles were washed by PBS for 10 min and permeabilized by Triton X-100 for 30 min at room temperature. The muscles were then incubated with primary antibody α-Bungarotoxin (B35451) targeting nicotinic acetylcholine (ACh) receptor overnight. The samples were mounted after two times wash of PBS. Fluorescent images were taken by inverted multiphoton laser-scanning microscope (FV1200MPE, Olympus) and analyzed and quantified by Image J based on established protocol (5). In brief, using Image J software, binary images and skeleton images were generated from original images (Supplementary Figure 2B). From binary images, "AChR cluster area" was calculated as the standing area per island. From skeleton images, "Branching" was calculated as the number of pixels with three neighbors. "Discontinuity" was calculated as the number of pixels which represent the end of a line of pixels with one neighbor.
To stain the cultured cells, the adherent cells were fixed in 4% PFA for 20 minutes at room

temperature, then permeabilized by 0.1% Triton X-100. After blocking with 5% BSA, cells were incubated with 1:100 anti-MYOD (Abcam, ab64159), 1:100 anti-PAX7 (Abcam, ab199010), 1:100 anti-LC3-β (CST, 2775), 1:200 MHC IIa (SC-71) (DSHB, IA, USA). After probed with secondary inflorescent antibody, cells were counterstained by DAPI and then mounted with Prolong®Gold Antifade Reagent (Invitrogen). For MHC staining of myofibers, the fluorescent area was used to evaluate fiber area and the nucleus number of a single fiber was used to evaluate the nucleus fusion capacity using Image J software.

Intracellular Cl^- and Ca^{2+} measurement

The PMBs seeded on 25 mm cover slips at a confluence of 40%. The medium was changed to differentiation when the cells reach confluent and cultured for 4 days to test the differentiated myotubes. Cells were washed by Margo bath three times. Incubate at 37°C for 30 min after addition of 10 mM N-(6-methoxyquinolyl)-acetoethyl ester (MQAE, Invitrogen) or 5μM Fura-2 (Thermo Fisher) that can label Cl^- or Ca^{2+} by fluorescence. The concentration of intracellular Cl^- is negatively correlated with the intensity of fluorescent intensity. Intracellular Ca^{2+} change was measured by the ratio of 340/380 fluorescent intensity. The image capture was performed by Nikon fluorescent microscope (Eclipse Ti, Nikon, Tokyo, Japan) every 3 sec. The MQAE loss was used to evaluate the inhibition efficacy of CFTRinh-172 in PMBs isolated from wild-type and DF508. The change of F340/380 after treatment was used to evaluate the effect of ACh (1mM, Sigma), and KCl (60mM, Sigma).

RNA extraction and qPCR

Total RNA was extracted with RNAiso Plus reagent (TaKaRa) following the manufacturer's protocol. After extraction, cDNAs were reverse transcribed from RNA using PrimeScript RT Reagent Kit with gDNA Eraser (TaKaRa). Power SYBR Green PCR Master Mix (Takara) was used to perform the qPCR to detect the mRNA. The qPCR reaction was performed in ABI +-QuantStudio 12 Flex Real-Time RCR system (Thermo Fisher Scientific). The relative fold changes of candidate genes were calculated by using the $2^{-\Delta\Delta Ct}$ method. The primers for qPCR were included in Supplementary table 2.

Transmission electron microscopy (TEM)

Gastrocnemius muscle was rinsed in 0.1M pH7.2 Sorensen phosphate and fixed in 2.5% glutaraldehyde for 30min. Then the tissue was cut into 1-3mm small pieces and further fixed over nights at 4°C, postfixed in 1% Osmium tetroxide for 1 hour at 4°C, dehydrated, and embedded by Spurr Low-Viscosity Embedding Kit (Sigma, EM0300) at either a longitudinal or transverse orientation. The embedded tissues were then trimmed to a 70-nm thickness using EM UC7 Ultramicrotome (Leica, Germany) and mounted onto a copper grid (Electron Microscope Sciences, EMS200-Cu) and contrasted by UranyLess EM Stain (EMS 22409), and 0.5% lead citrate, and observed with electron microscope (Model H-7650, Hitachi, Japan) at 80 kV. The indicated organelle area was quantified by image J from randomly selected 18 fields. For the measurement of mitophagosome, the number of representative mitophagy phenotype was recorded in total 10 randomly selected images.

Reverse Transcription PCR (RT-PCR) and Sanger sequencing

The RNA extraction and reverse transcription were performed based on the last section. PCR was performed using PrimeScript™ One-Step RT-PCR Kit (RR055A, Takara) based on the protocol: 5 min denaturation at 94°C, 35 cycles of 94°C for 1 min, 55°C for 1 min, and 72°C for 1 min. The DNA products were purified from an agarose gel with the PureLink Quick Gel extraction kit (Invitrogen). Then, the samples were sent to BGI (Hong Kong) for sanger sequencing. The sequencing results were aligned by Standard Nucleotide BLAST (PubMed).

Cell proliferation assay

Cell proliferation ability was measured by Cell Counting Kit-8 (CCK-8) kit (ab228554, Abcam). Briefly, cell suspension with 8000 cells/mL was seeded into the 96-well plate (0.1 mL per well) and incubated at 37°C and 5% CO^2. After 48 hours of incubation, cells were treated with CCK-8 solution (10 μL) and held in the incubator for 3-4 hours. The statistical quantification was performed at the absorbance rate of OD 450 nm.

References

1. Tumasian, R.A., A. Harish, G. Kundu, J.-H. Yang, C. Ubaida-Mohien, M. Gonzalez-Freire, M. Kaileh, L.M. Zukley, C.W. Chia, and A. Lyashkov, Skeletal muscle transcriptome in healthy aging. *Nature Communications*, 2021; 12(1): p. 1-16.
2. Bloemberg, D. and J. Quadrilatero, Rapid determination of myosin heavy chain expression in rat, mouse, and human skeletal muscle using multicolor immunofluorescence analysis. *PloS one*, 2012; 7(4): p. e35273.
3. Long, D.E., A.G. Villasante Tezanos, J.N. Wise, P.A. Kern, M.M. Bamman, C.A. Peterson, and R.A. Dennis, A guide for using NIH Image J for single slice cross-sectional area and composition analysis of the thigh from computed tomography. *PloS one*, 2019; 14(2): p. e0211629.
4. Wing-Hoi, C., L. Wing-Sze, Q. Ling, T. Ning, V.W.-Y. Hung, and L. Kwok-Sui, Type IIB human skeletal muscle fibers positively correlate with bone mineral density irrespective to age. *Chinese medical journal*, 2010; 123(21): p. 3009-3014.
5. Pratt, S.J., S.R. Iyer, S.B. Shah, and R.M. Lovering, *Imaging analysis of the neuromuscular junction in dystrophic muscle*, in *Duchenne Muscular Dystrophy*. 2018, Springer. p. 57-72.

Supplementary Table 1. Information of human subjects

No.	Gender	Age	Reason for surgery
1	F	7	Femoral fracture
2	M	8	Femoral fracture
3	F	18	Femoral fracture
4	M	26	Femoral fracture
5	M	29	Hip fracture
6	F	38	Femoral fracture
7	F	41	Femoral fracture
8	F	49	Hip replacement
9	F	52	Hip fracture
10	M	54	Femoral fracture
11	M	59	Hip replacement
12	F	59	Femoral fracture
13	F	62	Hip fracture
14	F	64	Femoral fracture
15	M	66	Femoral fracture
16	M	67	Hip fracture
17	M	68	Femoral fracture
18	F	71	Femoral fracture
19	F	72	Hip replacement
20	F	74	Hip replacement
21	F	81	Femoral fracture
22	M	84	Hip fracture
23	F	86	Femoral fracture

Supplementary Table 2. Sequences of primers

H: human genes, M: mouse genes, F: forward, R: reverse.

Genes	Primer sequences
H-CFTR-F	GTGTGATTCCACCTTCTCCAA
H-CFTR-R	GCCTGGCACCATTAAAGAAA
M-Pgc1-b-F	CTTGGCTGCGCTTACGAAGA
M-Pgc1-b-R	GAAAGCTCGTCCACGTCAGAC
M-Nrf-1-F	AGCACGGAGTGACCCAAAC
M-Nrf-1-R	TGTACGTGGCTACATGGACCT
M-Nrf-2-F	TAGATGACCATGAGTCGCTTGC
M-Nrf-2-R	GCCAAACTTGCTCCATGTCC
M-Tfam-F	ATTCCGAAGTGTTTTTCCAGCA
M-Tfam-R	TCTGAAAGTTTTGCATCTGGGT
M-Mfn2-F	CTGGGGACCGGATCTTCTTC
M-Mfn2-R	CTGCCTCTCGAAATTCTGAAACT
M-Mfn1-F	ATGGCAGAAACGGTATCTCCA
M-Mfn1-R	CTCGGATGCTATTCGATCAAGTT
M-Fis1-F	TGTCCAAGAGCACGCAATTTG
M-Fis1-R	CCTCGCACATACTTTAGAGCCTT
M-Bnip3-F	TCCTGGGTAGAACTGCACTTC
M-Bnip3-R	GCTGGGCATCCAACAGTATTT
M-Foxo3-F	CTGGGGGAACCTGTCCTATG
M-Foxo3-R	TCATTCTGAACGCGCATGAAG
M-Mafbx-F	CAGCTTCGTGAGCGACCTC
M-Mafbx-R	GGCAGTCGAGAAGTCCAGTC
M-Murf1-F	CCAGGCTGCGAATCCCTAC
M-Murf1-R	ATTTTCTCGTCTTCGTGTTCCTT
M-Lamp-2-F	TGTATTTGGCTAATGGCTCAGC
M-Lamp-2-R	TATGGGCACAAGGAAGTTGTC
M-Caspase-3-F	ATGGAGAACAACAAAACCTCAGT
M-Caspase-3-R	TTGCTCCCATGTATGGTCTTTAC
M-Caspase-9-F	TCCTGGTACATCGAGACCTTG
M-Caspase-9-R	AAGTCCCTTTCGCAGAAACAG
M-Apaf-1-F	AGTAATGGGTCCTAAGCATGTTG
M-Apaf-1-R	GCGATTGGGAAAATCACGTAAAA
M-Bcl2-F	ATGCCTTTGTGGAACTATATGGC
M-Bcl2-R	GGTATGCACCCAGAGTGATGC
M-Myod-F	CCACTCCGGGACATAGACTTG
M-Myod-R	AAAAGCGCAGGTCTGGTGAG
M-Myf5-F	CACCACCAACCCTAACCAGAG
M-Myf5-R	AGGCTGTAATAGTTCTCCACCTG

M-Myog-F	GAGACATCCCCCTATTTCTACCA
M-Myog-R	GCTCAGTCCGCTCATAGCC
M-Myf6-F	AGAGGGCTCTCCTTTGTATCC
M-Myf6-R	CTGCTTTCCGACGATCTGTGG
M-Atg5-F	TGTGCTTCGAGATGTGTGGTT
M-Atg5-R	GTCAAATAGCTGACTCTTGGCAA
M-Atg7-F	CATCCCTCTAATCCGGGGAC
M-Atg7-R	TCGGCTCGACACAGATCATCA
M-Atg12-R	AGGTCTGTAGTCGCGGAGAA
M-Atg12--F	CGGGAACACCAAGTTTCACT
M-Lc3b-F	TTATAGAGCGATACAAGGGGGAG
M-Lc3b-R	CGCCGTCTGATTATCTTGATGAG
M-Gapdh-F	AACGACCCCTTCATTGAC
M-Gapdh-R	TCCACGACATACTCAGCAC
M-Atg6-F	ATGGAGGGGTCTAAGGCGTC
M-Atg6-R	TCCTCTCCTGAGTTAGCCTCT
M-Pax7-F	GCTCAGAATCAAGTTCGGGA
M-Pax7-R	TGATTCCACATCTGAGCCCT
M-Cftr-F	GCTGACACTTTGCTTGCCCTGAG
M-Cftr-R	GCTTGCTGATGGTCGACATAGGG
M-Il-6-F	CCAAGAGGTGAGTGCTTCCC
M-Il-6-R	CTGTTGTTCAGACTCTCTCCCT
M-inos-F	GTTCTCAGCCCAACAATACAAGA
M-inos-R	GTGGACGGGTCGATGTCAC
M-Pgc1a-F	TATGGAGTGACATAGAGTGTGCT
M-Pgc1a-R	CCACTTCAATCCACCCAGAAAG
H-GAPDH-F	AGGGTCATCATCTCTGCC
H-GAPDH-R	CCATCACGCCACAGTTTC
M-Bcl2l13-F	ATGGCGTCCTCTACGACTG
M-Bcl2l13-R	GGTGAGGGACCTTGTTGTTTC
M-Atg12-F	AGGTCTGTAGTCGCGGAGAA
M-Atg12-R	CGGGAACACCAAGTTTCACT
M-Pink-F	TGCTGAAACTGCCTTCCTATCA
M-Pink-R	TGCTGAAACTGCCTTCCTATCA
M-Parkin-F	GAGGTCGATTCTGACACCAGC
M-Parkin-R	CCGGCAAAAATCACACGCAG
M-Drp1-F	CAGGAATTGTTACGGTTCCCTAA
M-Drp1-R	CCTGAATTAACTTGTCCCGTGA
M-Tnfa-F	CCTGTAGCCCACGTCGTAG
M-Tnfa-R	GGGAGTAGACAAGGTACAACCC
M-Cxcr5-F	TGGCCTTCTACAGTAACAGCA
M-Cxcr5-R	TGGCCTTCTACAGTAACAGCA

Supplementary Fig.S1. Age-dependent skeletal muscle defects in CFTR mutant (DF508) mice

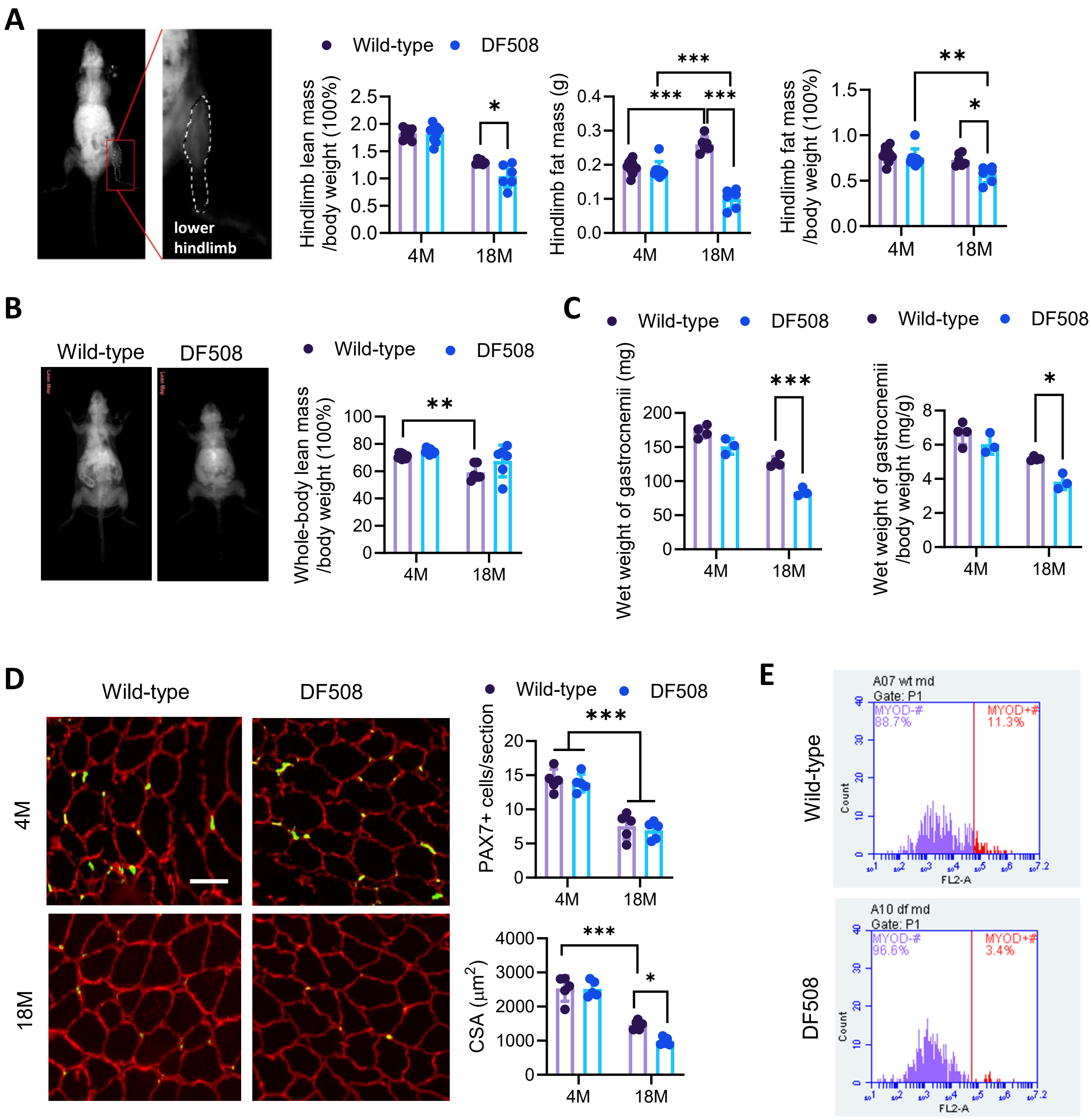

A-B) Dual-energy X-ray absorptiometry (DEXA) analysis of lean or fat mass in lower hindlimb (A) or whole-body (B) in male wild-type or DF508 mice at 4- and 18- month-old (4M and 18M). **p*<0.05, ***p*< 0.01, ****p*<0.001, Two-way ANOVA with Bonferroni's multiple comparison test. n = 6-10. Representative DEXA images are shown on the left. **C)** Wet weight of gastrocnemii from male wild-type or DF508 mice. ***p*<0.01, Two-way ANOVA with Bonferroni's multiple comparison test. n = 4. **D)** Representative images of laminin (red) and PAX7 (green) staining in gastrocnemii from male wild-type or DF508 mice with quantification of PAX7 positive cells and myofiber cross-sectional area (CSA). **p*<0.05, ****p*<0.001, Two-way ANOVA with Bonferroni's multiple comparison test. n = 5. Scale bar, 20μm. **E)** Flow cytometry analysis of MYOD-positive cells isolated from gastrocnemii of male wild-type and DF508 mice.

Supplementary Fig.S2. Neuromuscular junction function test in DF508 mice

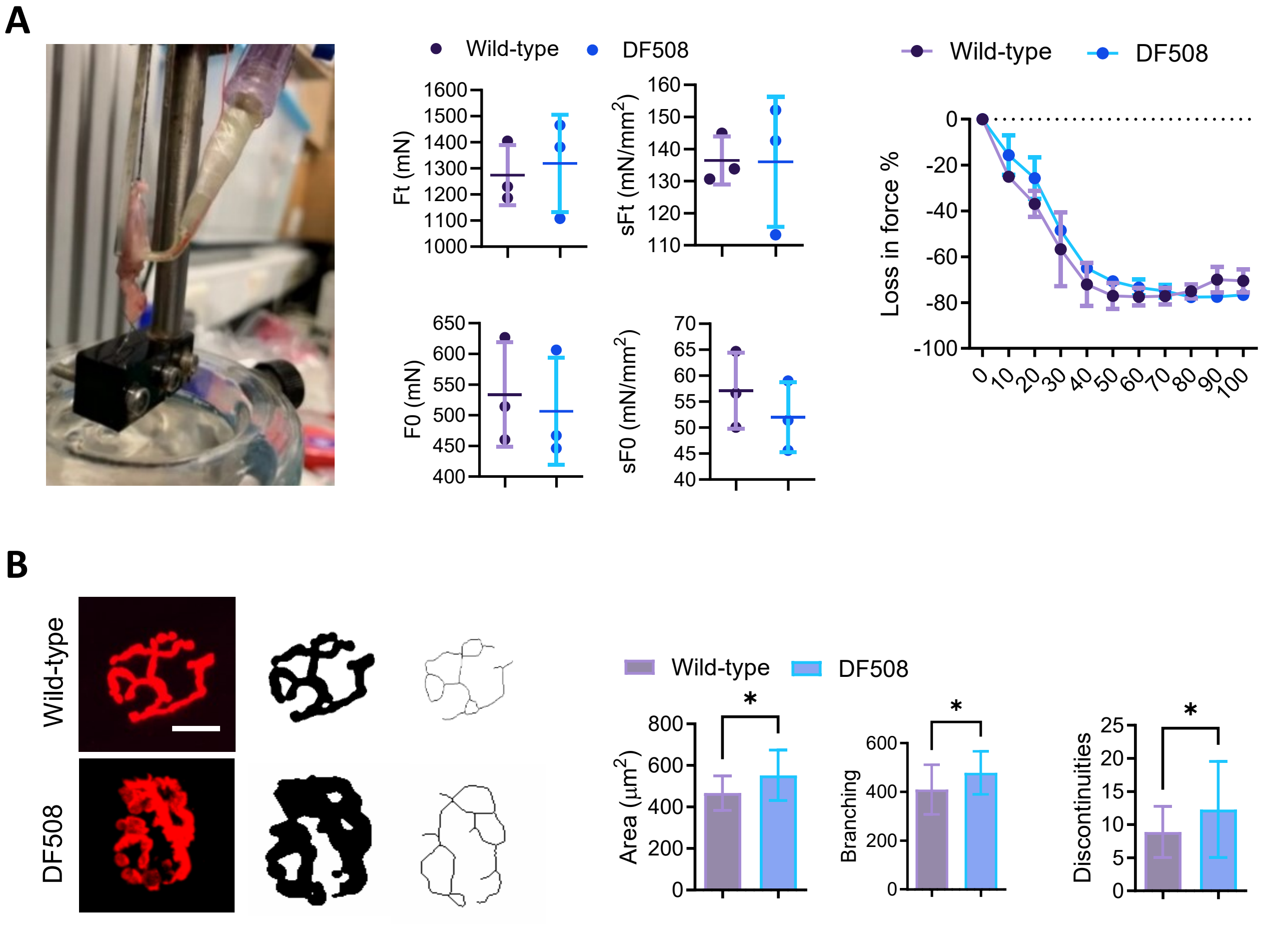

A) *Ex vivo* neuromuscular junction functional test of female wild-type or DF508 mice at 20-month-old. *Left:* Photograph of an isolated mouse sciatic nerve-triceps surae specimen in the contraction measurement. *Middle*: quantification of tetanic force (Ft) and twitch force (F0). *Right*: linear plots of neurotransmission failure after fatigue stimulation. Unpaired t-test. n = 3.

B) Representative images of AChR staining in extensor digitorum longus from female wild-type or DF508 mice at 20-month-old with quantification of area, branching, and discontinuities. *p<0.05, unpaired t-test. n = 3. Scale bar, 10mm.

Supplementary Fig.S3. Involvement of CFTR in myogenic differentiation *in vitro*

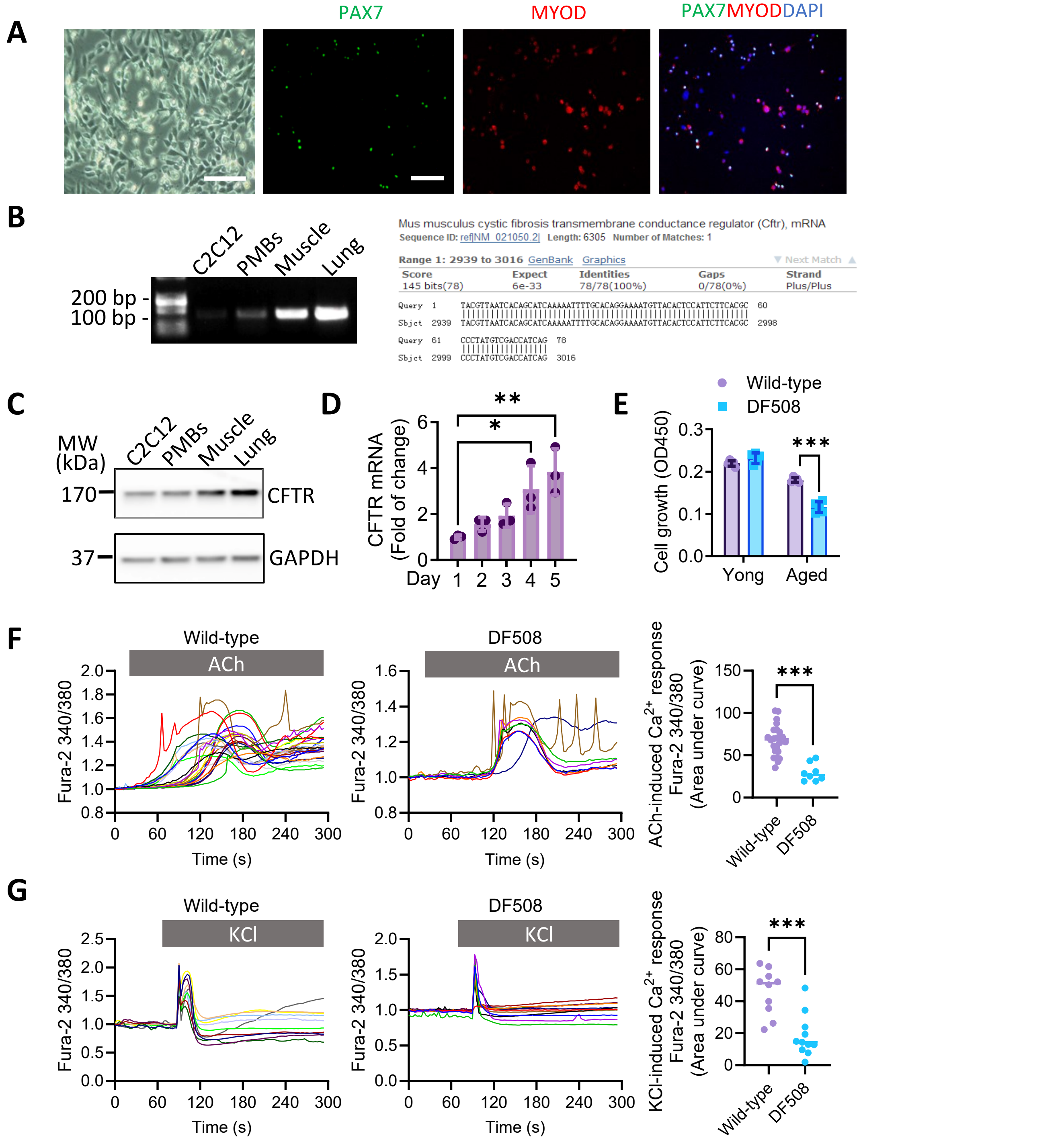

A) Bright-field or immunofluorescence images for PAX7 (green) and MYOD (red) in mouse primary myoblasts (PMBs). Scale bar, 50μm. **B-C)** Reverse transcription (RT) PCR (B) and western blots (C) for CFTR in C2C12 cells, PMBs and gastrocnemius or lung tissues from male mice at 4-month-old. *Right*: Sanger sequencing of RT-PCR product in C2C12 cells aligned by Standard Nucleotide BLAST in NCBI. GAPDH was used as a loading control in C. **D)** qPCR analysis of CFTR mRNA levels in PMBs at day 1 to 5 after myogenic differentiation was induced *in vitro*. *$p<0.05$, **$p<0.01$, One-way ANOVA with Bonferroni's multiple comparison test. n = 3. **E)** CCK8 cell-proliferation assay (48 hours after seeding) of PMBs from male wild-type or DF508 mice at 2- (young) or 14- (aged) month-old. ***$p<0.001$, Two way ANOVA with Bonferroni's multiple comparison test. n = 5. **F-G)** Real-time measurement of intracellular Ca^{2+} level in wild-type or DF508 PMBs when acetylcholine (F, ACh, 1 mM, n = 8-24) or KCl (G, 60 mM, n = 10-11) were added into the bath. ***$p<0.001$, unpaired t-test.

Supplementary Fig.S4. Effect of CFTR knockdown on myogenic differentiation in vitro

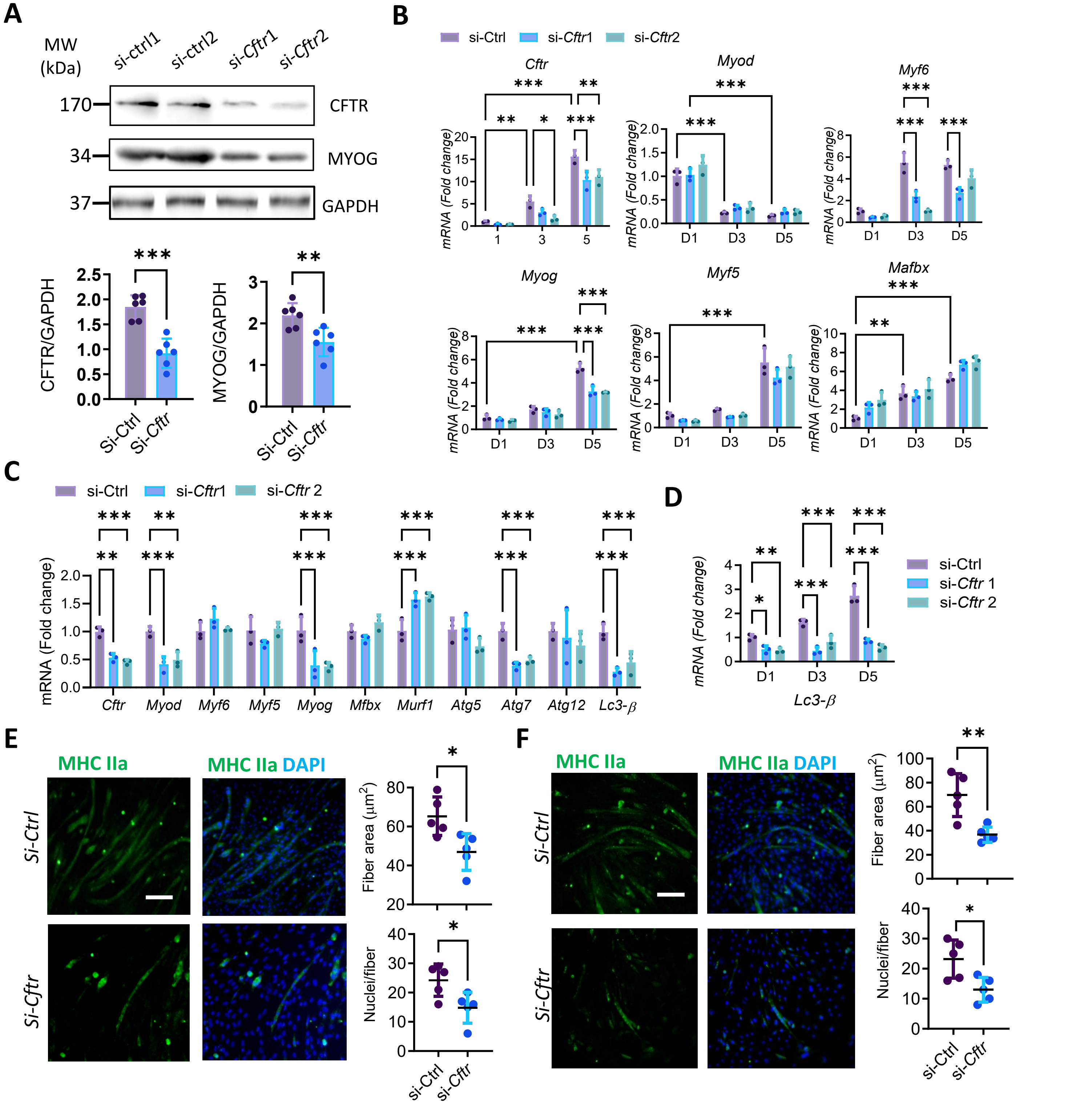

A) Western blots for CFTR and MYOG in PMBSs treated with siRNAs targeting CFTR (si-*Cftr*) or nontargeting control (si-Ctrl) at day 5 after myogenic differentiation was induced *in vitro* (see methods). $^{**}p<0.01$, $^{***}p<0.001$, unpaired t-test. n = 6.

B-F) Quantitative PCR (qPCR) for CFTR, myogenesis-, atrophy- or autophagy-related genes (B, C and D) and immunofluorescence staining for MHC IIa (E and F) in PMBs (B,C and E) and C2C12 (D and F) at day 3-5 post the myogenic differentiation induction treated with si-Ctrl or si-*Cftr*. DAPI was used to label nuclei, the number of which were counted. MHC IIa-positive area was quantified as fiber area. Scale bar, 50μm. $^{*}p<0.05$, $^{**}p<0.01$, $^{***}p<0.001$, One-way (C) or Two-way ANOVA (B and D) with Bonferroni's multiple comparison test, and unpaired t-test (E and F). n = 3-5.

Supplementary Fig.S5. Mitochondria and autophagosome changes in DF508 mice

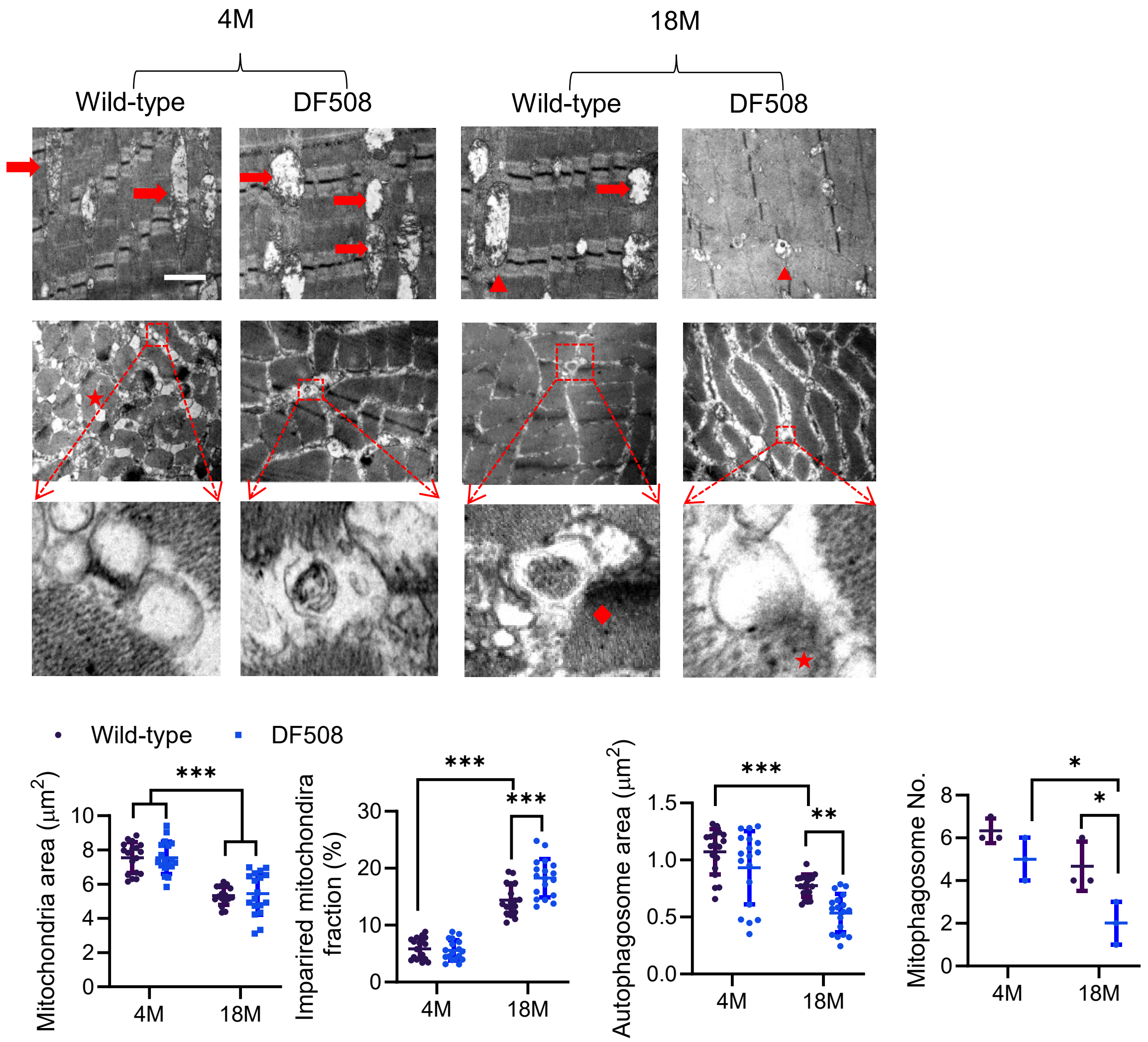

Representative transmission electron microscopy (TEM) images of the intermyofibrillar mitochondria and lysosomal vesicles in gastrocnemii from male wild-type or DF508 mice at 4- and 18-month-old (4M or 18M) with quantification of mitochondrial area (arrows), impaired mitochondria (arrowheads), autophagosome area (asterisk), and mitophagosome (rhombus). *$p<0.05$, **$p<0.01$, ***$p<0.001$, Two-way ANOVA with Bonferroni's multiple comparison test. n = 3. Scale bar, 100nm.

Supplementary Fig.S6. Effect of adeno-virus mediated overexpression of CFTR on skeletal muscles in aged mice

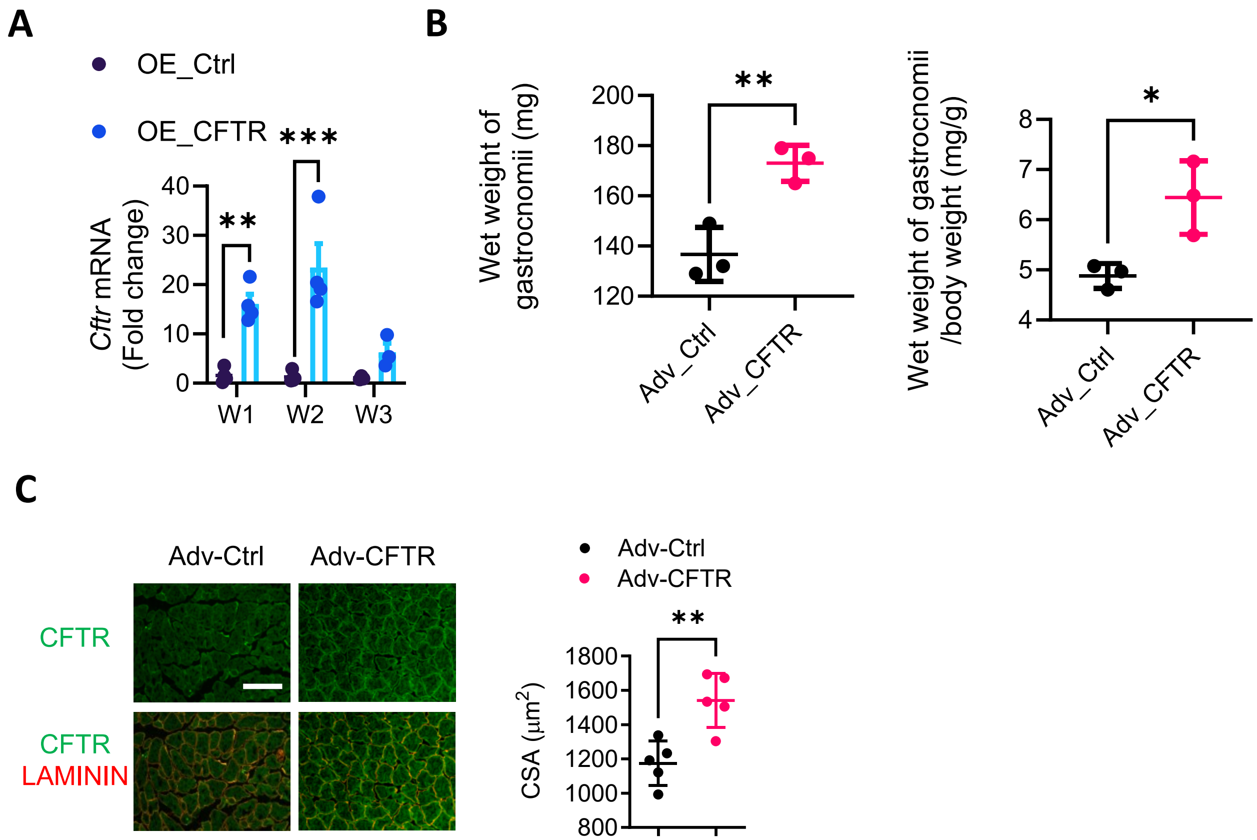

A) qPCR analysis of CFTR mRNA level in gastrocnemii from 15-month-old female wild-type mice at week 1, 2 or 3 (W1, W2, or W3) after injected with adenoviruses containing human CFTR gene (Adv-CFTR) or control viruses (Adv-Ctrl). *$p<0.05$, **$p<0.01$, ***$p<0.001$, Two-way ANOVA with Bonferroni's multiple comparison test. n = 3-4. **B-C)** Wet weight (B) and immunofluorescence staining (C) for CFTR (green) and laminin (red) in the Adv-CFTR- or Adv-Ctrl-treated gastrocnemii collected at W2. Scale bar, 50μm. *$p<0.05$, **$p<0.01$, unpaired t-test. n = 3-5.

Supplementary Fig.S7. Effect of VX809 on myoblasts in vitro

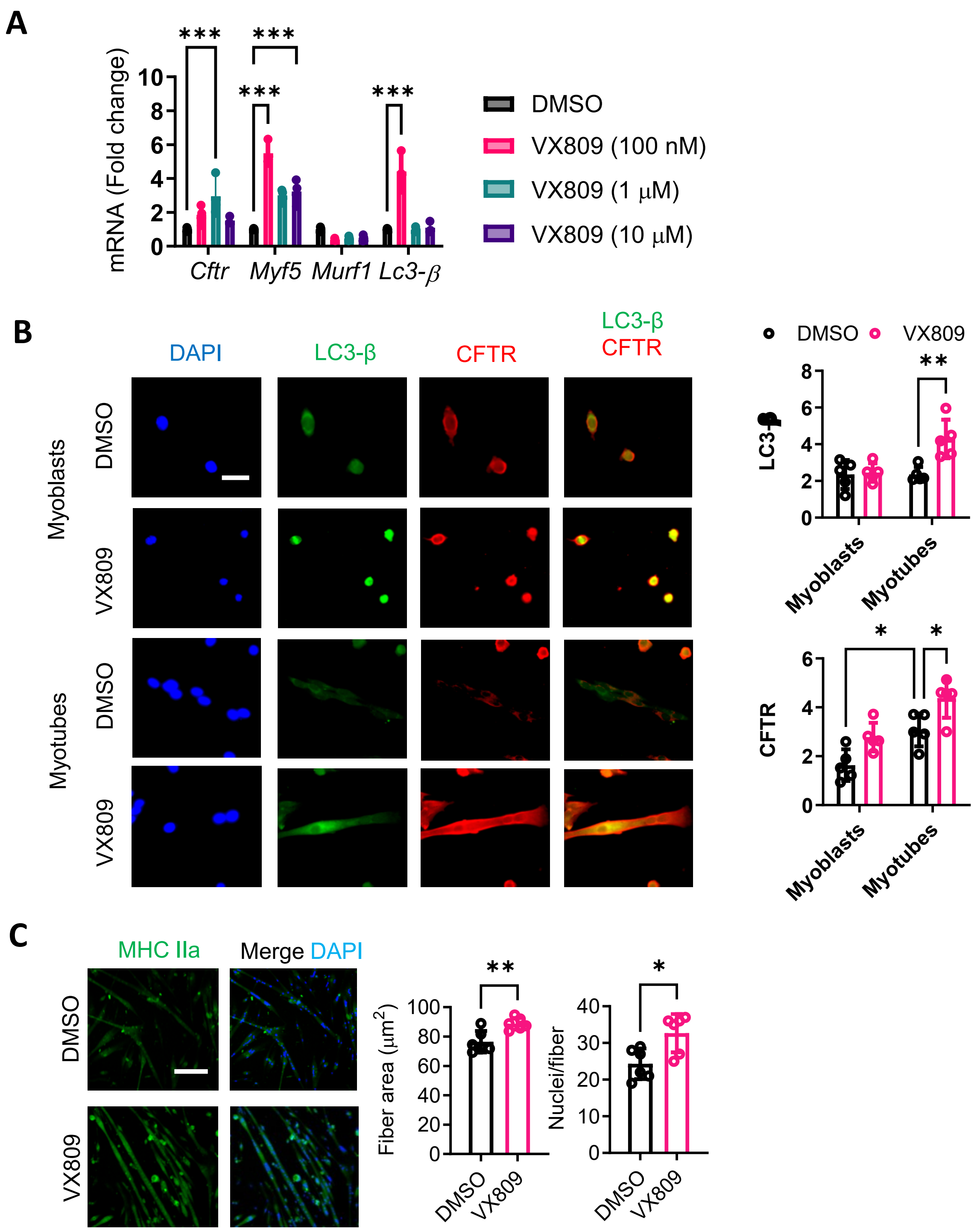

A) qPCR analysis of *Myf5*, *Murf1*, and *Lc3-β* in PMBs treated with VX809 or DMSO as control at day 3 post myogenic differentiation induction ***p<0.001, Two-way ANOVA with Bonferroni's multiple comparison test. n = 3.

B-C) Representative images of immunofluorescence staining for CFTR, LC3-β (B) and MHC IIa (C) in undifferentiated PMBs or differentiated myotubes treated with VX809 (100 nM) or DMSO as control, with quantification of the fluorescent density (B), fiber area and nuclei/fiber (C). *p<0.05, **p<0.01, Two-way ANOVA with Bonferroni's multiple comparison test (B) or unpaired t-test (C), n = 5-6. Scale bars, 50μm.

Supplementary Fig.S8. Effect of VX809 on skeletal muscles in aged mice

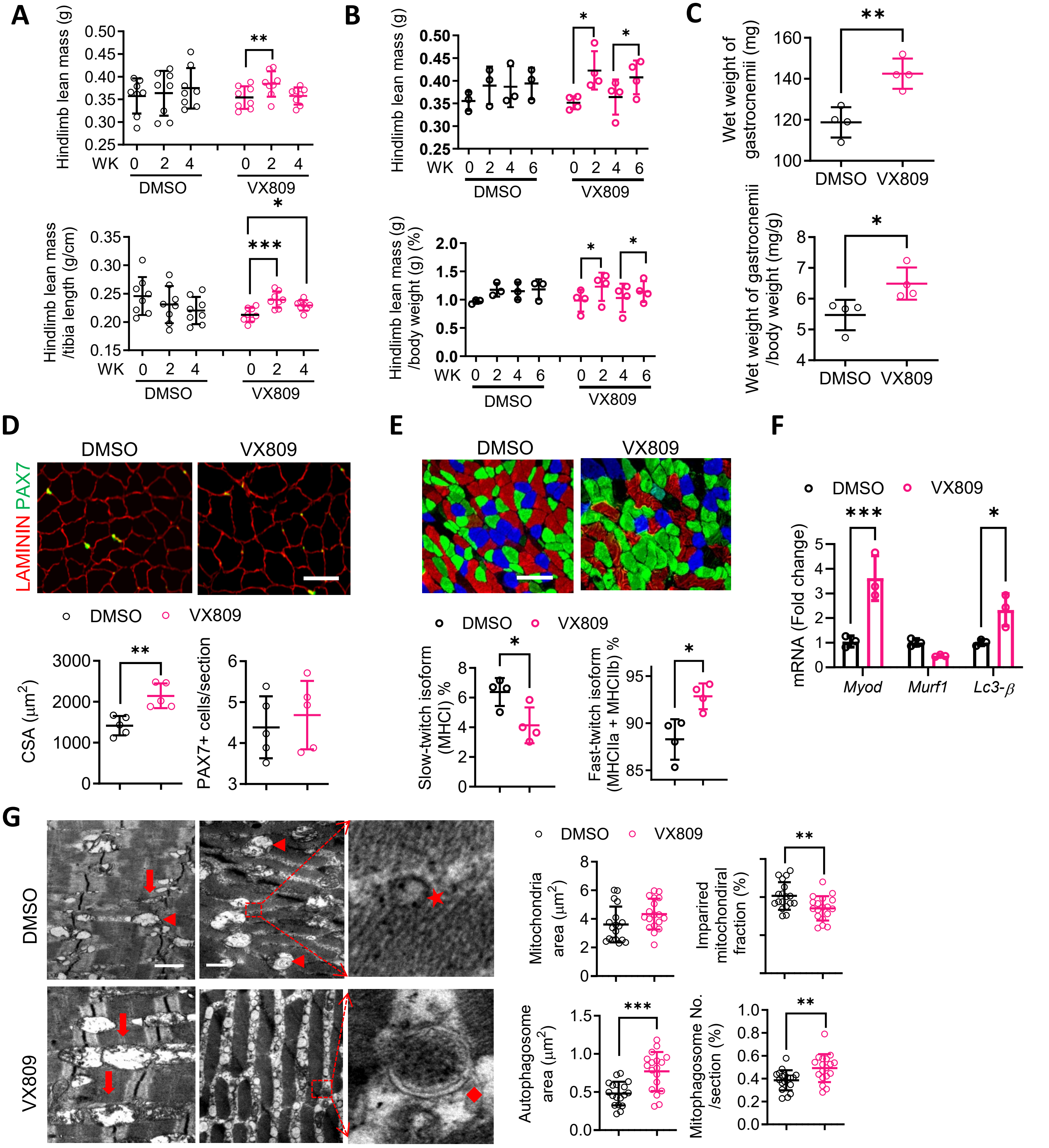

A-B) Lean mass (by DEXA) of lower hindlimb in female 29-month-old (A) or male 16-month-old (B) wild-type mice injected with VX809 or DMSO at week 0, 2, 4, and 6 post the injection with or without normalization to the length of tibia (A) or body weight (B). **p*<0.05, ***p*<0.01, ****p*<0.001, paired t-test. n = 3-8. **C-D)** Wet weight (C) and immunofluorescence staining for laminin (red) and PAX7 (green) with quantification of PAX7 positive cells and myofiber CSA (D) of gastrocnemii injected with DMSO or VX809 collected at week 4 post the injection in female wild-type mice at 29-month-old. Scale bar, 20μm. **p*<0.05, ***p*<0.01, unpaired t-test. n = 3-5. **E-F)** Representative images of MHC staining with quantification of slow- or fast- twitch fibers (E) and qPCR of *Myod*, *Murf-1*, and *Lc3-β* (F) in gastrocnemii injected with DMSO or VX809 collected at week 2 post the injection in female wild-type mice at 16-month-old. Scale bar, 20μm. **p*<0.05, ****p*<0.001, unpaired t-test. n = 3-5. **G)** Representative TEM images of the intermyofibrillar mitochondria and lysosomal vesicles in gastrocnemii injected with DMSO or VX809 collected at week 2 in male wild-type mice at 16-month-old with quantification of mitochondrial area (arrows), impaired mitochondria (arrowheads), autophagosome area (asterisk), and mitophagosome (rhombus). ***p*<0.01, ****p*<0.001, unpaired t-test. n = 3. Scale bars, 100nm.
